# Intertumoral Differences Dictate the Outcome of TGF-β Blockade on the Efficacy of Viro-Immunotherapy

**DOI:** 10.1158/2767-9764.CRC-23-0019

**Published:** 2023-02-23

**Authors:** Christianne Groeneveldt, Jurriaan Q. van Ginkel, Priscilla Kinderman, Marjolein Sluijter, Lisa Griffioen, Camilla Labrie, Diana J.M. van den Wollenberg, Rob C. Hoeben, Sjoerd H. van der Burg, Peter ten Dijke, Lukas J.A.C. Hawinkels, Thorbald van Hall, Nadine van Montfoort

**Affiliations:** 1 Department of Medical Oncology, Oncode Institute, Leiden University Medical Center, Leiden, the Netherlands.; 2 Department of Gastroenterology and Hepatology, Leiden University Medical Center, Leiden, the Netherlands.; 3 Department of Cell and Chemical Biology, Leiden University Medical Center, Leiden, the Netherlands.; 4 Department of Cell and Chemical Biology, Oncode Institute, Leiden University Medical Center, Leiden, the Netherlands.

## Abstract

The absence of T cells in the tumor microenvironment of solid tumors is a major barrier to cancer immunotherapy efficacy. Oncolytic viruses, including reovirus type 3 Dearing (Reo), can recruit CD8^+^ T cells to the tumor and thereby enhance the efficacy of immunotherapeutic strategies that depend on high T-cell density, such as CD3-bispecific antibody (bsAb) therapy. TGF-β signaling might represent another barrier to effective Reo&CD3-bsAb therapy due to its immunoinhibitory characteristics. Here, we investigated the effect of TGF-β blockade on the antitumor efficacy of Reo&CD3-bsAb therapy in the preclinical pancreatic KPC3 and colon MC38 tumor models, where TGF-β signaling is active. TGF-β blockade impaired tumor growth in both KPC3 and MC38 tumors. Furthermore, TGF-β blockade did not affect reovirus replication in both models and significantly enhanced the Reo-induced T-cell influx in MC38 colon tumors. Reo administration decreased TGF-β signaling in MC38 tumors but instead increased TGF-β activity in KPC3 tumors, resulting in the accumulation of α-smooth muscle actin (αSMA^+^) fibroblasts. In KPC3 tumors, TGF-β blockade antagonized the antitumor effect of Reo&CD3-bsAb therapy, even though T-cell influx and activity were not impaired. Moreover, genetic loss of TGF-β signaling in CD8^+^ T cells had no effect on therapeutic responses. In contrast, TGF-β blockade significantly improved therapeutic efficacy of Reo&CD3-bsAb in mice bearing MC38 colon tumors, resulting in a 100% complete response. Further understanding of the factors that determine this intertumor dichotomy is required before TGF-β inhibition can be exploited as part of viroimmunotherapeutic combination strategies to improve their clinical benefit.

**Significance:**

Blockade of the pleiotropic molecule TGF-β can both improve and impair the efficacy of viro-immunotherapy, depending on the tumor model. While TGF-β blockade antagonized Reo&CD3-bsAb combination therapy in the KPC3 model for pancreatic cancer, it resulted in 100% complete responses in the MC38 colon model. Understanding factors underlying this contrast is required to guide therapeutic application.

## Introduction

Oncolytic viruses (OV) are increasingly recognized as potent anticancer agents due to their preferential infection of cancerous cells and stimulation of host antitumor immunity (1). The mammalian reovirus type 3 Dearing strain (T3D) is one of the most prominent OVs under clinical evaluation and displays an excellent safety record in clinical trials (2, 3). Reoviruses show an inherent preference for replication in and lysis of transformed, but not healthy cells (4). Although reovirus has demonstrated moderate antitumor efficacy as monotherapy (5, 6), studies have shown that its potential might be better utilized as a part of combinatorial approaches (7). For example, we recently demonstrated that sensitizing the tumor microenvironment (TME) of murine pancreatic KPC3 tumors with reovirus significantly enhanced the efficacy of otherwise noneffective CD3-bispecific antibodies (CD3-bsAb). This enhanced efficacy could be attributed to the capability of reovirus to induce a fast IFN response which was followed by a potent influx of CD8^+^ T cells (8). Others have shown that reovirus can sensitize the TME for immune checkpoint inhibition by enhancing the intratumoral density of tumor-specific CD8^+^ T cells and upregulating immune checkpoint inhibitor programmed death-ligand 1 (PD-L1) expression (9).

Although the use of OVs is very promising to attract T cells to solid tumors and improve the efficacy of immunotherapeutic strategies, these combination approaches rarely lead to complete cures. Various tumor types such as colorectal cancer, ovarian cancer, and pancreatic ductal adenocarcinoma (PDAC; refs. 10–12) often present with high TGF-β signaling, which might be another barrier to effective combinatorial immunotherapy (13–15). TGF-β acts as a tumor-promoting cytokine by stimulating cancer cell migration and invasion, extracellular matrix remodeling, epithelial-to-mesenchymal transition (EMT), and the induction of an immunosuppressive TME (16). In particular, TGF-β acts as an immunosuppressive factor by inhibiting the generation and function of CD4^+^ and CD8^+^ effector T cells and dendritic cells, while promoting the expansion of regulatory T cells (Treg) and myeloid-derived suppressor cells (17, 18). Indeed, TGF-β blockade can promote expansion of CD8^+^ T cells, reduce the level of Tregs, and induce the polarization from protumorigenic M2 macrophages to antitumor M1 macrophages (19, 20). Altogether, these observations hint toward a potential beneficial effect of TGF-β inhibition on the efficacy of immunotherapeutic strategies. For example, TGF-β inhibition has increased the efficacy of checkpoint blockade in mouse models for mammary carcinoma and metastatic breast cancer, and colorectal cancer (21–24). We hypothesized that the reovirus-induced increase in intratumoral T cells, combined with TGF-β inhibition to remove the immunosuppressive barrier in the TME, would also strongly enhance the efficacy of viroimmunotherapeutic strategies. In the current study, we investigated whether inhibition of TGF-β signaling further enhanced the efficacy of reovirus and CD3-bsAb therapy in preclinical tumor models with high TGF-β signaling.

## Materials and Methods

### Reovirus

The wild-type (WT) reovirus strain R124 (further referred to as Reo) was previously isolated from a heterogeneous reovirus T3D stock (VR-824) obtained from the ATCC by two rounds of plaque purification using HER911 cells (RRID:CVCL_1K15; ref. 25). All experiments were performed using cesium chloride (CsCl)-purified stocks as described earlier (8). The total amount of particles was calculated on the basis of OD_260_ values where 1 OD equals 2.10 × 10^12^ reovirus particles/mL, and the infectious titer was quantified by plaque assay on HER911 cells.

### Cell Lines and Culture

The murine pancreatic cancer cell line KPC3 (RRID:CVCL_A9ZK; ref. 8) is a low-passage derivate of a primary KPC tumor with mutant *p53* and *K-ras* (26, 27) from a female C57BL/6 mouse. KPC3.TRP1 cells (RRID:CVCL_A9ZL) were generated as described (28) and selected for expression of tyrosine-related protein (TRP1) by cell sorting using an αTRP1 antibody (clone: TA99). The MC38 cell line (RRID: CVCL_B288; Kerafast, ENH204-FP) is a chemically-induced murine colon carcinoma and was obtained from Prof. F. Ossendorp (Leiden University Medical Center, Leiden, the Netherlands). MC38.TRP1 cells were generated as described before for KPC3.TRP1 (28) by transfection of MC38 cells with a *TRP1/gp75*-coding plasmid using lipofectamine (Invitrogen) in a 1:3 ratio. Transfected cells were selected with 400 μg/mL geneticin (G418, Thermo Fisher Scientific) and sorted twice for expression of TRP1 as described above. All cells were cultured at 37°C in a humidified atmosphere containing 5% CO_2_ in Iscove's Modified Dulbecco's Medium (Invitrogen) supplemented with 8% FCS (Bodinco), 2 mmol/L l-glutamine (Gibco), 100 μg/mL penicillin, and 100 μg/mL streptomycin (Gibco). Cell lines were assured to be free of *Mycoplasma* by regular PCR analysis. Authentication of the cell lines was done by short tandem repeat profiling (IDEXX BioAnalytics) and cells were passaged no more than six times before their use in experiments.

### Antibodies for *In Vivo* Administration

The CD3xTRP1 bsAb used is a knob-into-hole bispecific based on murine IgG2a with an Fc Silent mutation, featuring one arm with an anti-mouse CD3e single-chain variable fragment based on the clone 145-2C11, and the other arm containing the TA99 clone directed against TRP1 (bAb0136; Absolute Antibody). TGF-β blockade was performed using the monoclonal TGF-β-blocking antibody (clone 1D11.16.8; InVivoMAb anti-mouse/human/rat/monkey/ hamster/canine/bovine TGF-β1, 2, 3; BioXCell).

### Mouse Experiments

Male C57BL/6J mice (RRID:IMSR_JAX:000664; 6–8 weeks old) were purchased from Charles River Laboratories. Male nonobese diabetic (NOD).Cg-*Prkdc^scid^Il2rg^tm1Wjl^*/SzJ (NSG) mice (RRID:IMSR_JAX:005557; 6–8 weeks old) were obtained from The Jackson Laboratory. TGF-β receptor II (TβRII) knockout (KO) mice (TβRII^fl/fl^; ref. 29) were crossed with CD8a-driven Cre-knock-in mice (RRID:IMSR_JAX:008766) to generate CD8Cre^+/−^TβRII^fl/fl^ (CD8 TβRII KO) and CD8Cre^−/−^TβRII^fl/fl^ (TβRII WT) mice. Both male and female CD8 TβRII KO and TβRII WT mice (7–22 weeks old) were used in the experiment. Genomic PCR was conducted to analyze the genotypes of mice using ear DNA and gene-specific primers for the conditional TGF-βRII locus (29) and Cre construct (CRE transgene 5′-CAA TGG AAG GAA GTC GTG GT-3′; wt 5′-CAC ACA TGC AAG TCT AAA TCA GG-3′; CRE common 5′-TGG GAT TTA CAG GGC ATA CTG-3′).

All mouse experiments were individually prepared, reviewed, ethically approved, and registered by the institutional Animal Welfare Body of Leiden University Medical Center and carried out under project license AVD1160020187004, issued by the competent authority on animal experiments in the Netherlands (named CCD: Centrale Commissie Dierproeven). Power calculation was performed to define optimal sample size. Experiments were performed following the Dutch Act on Animal Experimentation and EU Directive 2010/63/EU (“On the protection of animals used for scientific purposes”) at the animal facility of the Leiden University Medical Center (LUMC), the Netherlands.

Mice were housed in individually ventilated cages with no more than 5 mice/cage. After 1 week of acclimatization after transport, mice were inoculated in the right flank with subcutaneous KPC3(.TRP1) tumors (1 × 10^5^ cells in 100 μL PBS/0.1% BSA) or MC38(.TRP1) tumors (5 × 10^5^ cells in 200 μL PBS/0.1% BSA). In the case of a rechallenge, mice that cleared the primary tumor were injected with the same amount of cells in the alternate flank. Intratumoral reovirus administration was performed under isoflurane anesthesia by injection of 1 × 10^7^ plaque-forming units (pfu) of reovirus or PBS as a control in a volume of 30 μL PBS. Intravenous administration of reovirus after tumor challenge was performed by injection of 1 × 10^8^ pfu of reovirus in a total volume of 100 μL PBS in the tail vein. Treatment with CD3xTRP1 bsAbs consisted of two to three intraperitoneal injections of 12.5 μg antibody in 100 μL PBS, given every other day. αTGF-β was administered 2–3×/week by intraperitoneal injections of 200 μg in 100 μL PBS.

Cages were randomly allocated to a certain treatment group by an independent researcher and treatments were given in a different order each time. During all experiments, tumors were measured three to five times a week in three dimensions using a caliper, in a blinded manner concerning the experimental group or genotype of the mice. For experiments where tumor growth was the experimental outcome, mice were sacrificed when the tumor volume exceeded 1,000 mm^3^. In the case where therapy response was determined: NR = no response; CR = complete response; and PR = partial response (regression or constant tumor volumes for at least 7 days). For interim blood analysis, blood was harvested by tail vein puncture. For intratumoral analysis experiments, mice were sacrificed at indicated days after treatment before tumors were collected. Tumors were divided into representative parts, which were either snap-frozen in liquid N2 and stored at −80°C for further analysis or fixed in 4% formaldehyde (AddedPharma) for IHC (see also Supplementary Methods 1). Alternatively, tumors were immediately processed to single cells suspensions for flow cytometry analysis.

### Cell Preparation and Flow Cytometry

Tumors were dissociated into a single-cell suspension as described before (8). Blood was incubated with red blood cell lysis buffer for 3 minutes at room temperature before use. Cells were incubated with Zombie Aqua Fixable Viability Dye (BioLegend) in PBS at room temperature followed by incubation with 2.4G2 FcR blocking antibodies (clone 2.4G2; BD Biosciences) in FACS buffer (PBS, 0.5% BSA and 0.2% NaN_3_) for 20 minutes on ice. If applicable, cells were incubated with Reo μ1_133–140_ tetramer conjugated to APC or the Rpl18 tetramer conjugated to PE (both generated in-house) for 1 hour at room temperature in FACS buffer, after which surface markers (Supplementary Table S1) were added directly to the tetramer mixture for 30 minutes of incubation at room temperature. For intracellular staining, cells were fixed and stained for transcription factors and nuclear proteins using the Foxp3/Transcription Factor Staining Buffer Set (eBiosciences) according to manufacturer's instructions. After completion of staining protocols, samples were fixed in 1% paraformaldehyde and acquired using a BD LSRFortessa X20 4L cell analyzer (BD Biosciences) at the Flow cytometry Core Facility (FCF) of LUMC in Leiden, the Netherlands (https://www.lumc.nl/research/facilities/fcf). Data were analyzed using FlowJo Software Version 10 (Becton, Dickinson, and Company).

### RNA Isolation and qRT-PCR

A representative snap-frozen proportion (10–30 mg) of each tumor or organ was disrupted in lysis buffer (Promega) using a stainless bead and the TissueLyser LT (Qiagen). Total RNA of *in vivo* samples was using the ReliaPrep RNA Tissue Miniprep System (Promega) according to the manufacturer's protocol. Total RNA from *in vitro* samples was isolated from cell pellets using the NucleoSpin RNA Kit (Macherey-Nagel) according to the manufacturer's instructions. A total of 500 ng of RNA was used to generate cDNA using the High-Capacity RNA-to-cDNA Kit (Thermo Fisher Scientific) according to the manufacturer's protocol. Reovirus genomic copies and expression levels of host genes (Supplementary Table S2) in tumors were measured by qRT-PCR as described previously (8). Reovirus S4 copy numbers were determined on the basis of a standard curve, generated with serial dilutions of plasmid pcDNA_S4. log_10_ S4 copy numbers were calculated using a previously described formula (30). The expression of host genes was normalized to reference genes *Mzt2* and *Ptp4a2* using the Bio-Rad CFX Manager 3.1 Software (Bio-Rad).

### Statistical Analysis

Sample size was calculated using the PS: Power and Sample Size Calculation program (Vanderbilt University, Nashville, TN; version 3.1.6; ref. 31). For experiments where tumor growth was the experimental read-out, mice were excluded when tumor engraftment was not successful (1% of all tumor engraftments). For qRT-PCR analysis, samples were excluded when RNA concentration and/or sample purity were too low. For flow cytometry data, tumor samples were excluded when evidence for draining lymph node contamination was present. All graphs were prepared and statistical analyses were performed using the GraphPad Prism software (version 8.0.2; RRID:SCR_002798). Statistical tests used for each figure are described in the figure legends. Significance levels are labeled with asterisks, with ns = nonsignificant; *, *P* < 0.05; **, *P* < 0.01; ***, *P* < 0.001; and ****, *P* < 0.0001.

### Data Availability

The data generated in this study are available upon reasonable request from the corresponding author.

## Results

### Early Blockade of TGF-β Signaling Delays Tumor Outgrowth of KPC3 and MC38 Tumors

**FIGURE 1:**
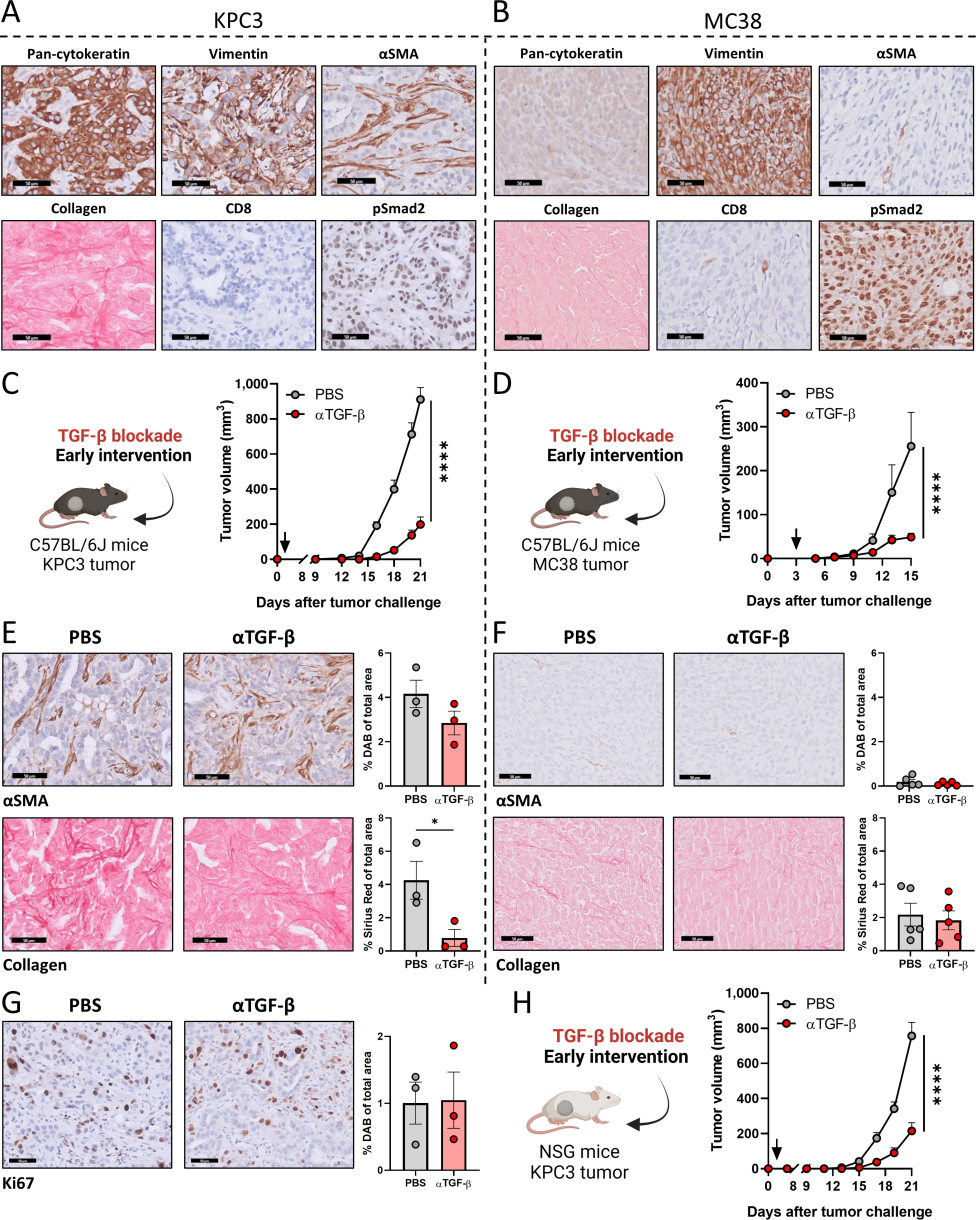
Early blockade of TGF-β signaling delays tumor outgrowth of KPC3 and MC38 tumors. Representative images obtained from IHC stainings of an untreated KPC3 (**A**) or MC38 (**B**) tumor for pan-cytokeratin, vimentin, αSMA, collagen, CD8, and phosphorylated Smad2 (pSmad2). Scale bars equal 50 μmol/L. Average tumor growth curves of immunocompetent KPC3 (**C**) or MC38 (**D**) tumor-bearing C57BL/6J mice (*n* = 5/group) after TGF-β blockade. Mice were subcutaneously engrafted with KPC3 cells (1 × 10^5^/mouse) or MC38 cells (5 × 10^5^/mouse) and received TGF-β-neutralizing antibodies (αTGF-β, 200 μg/injection every 3 days, starting from day 3 as indicated by the black arrow) as early intervention. IHC stainings for αSMA and collagen in representative KPC3 (**E**) or MC38 (**F**) tumors after indicated treatments. Scale bars, 50 μmol/L and stainings were quantified using ImageJ. **G,** IHC staining of Ki67 in KPC3 tumors treated with PBS or αTGF-β. Scale bars, 50 μmol/L and stainings were quantified using ImageJ. **H,** Average tumor growth curves of immunodeficient KPC3-bearing NSG mice (*n* = 8/group) after TGF-β blockade as early intervention, as described in C. Data represent mean ± SEM. Significance between PBS and αTGF-β in (E, F, and G) was determined using unpaired *t* tests. Significant differences in tumor growth between PBS and αTGF-β in (C, D, and H) were determined using an ordinary two-way ANOVA with Sidak multiple comparisons test. Significance levels: *, *P* < 0.05 and ****, *P* < 0.0001. Figures C, D and H were created with BioRender.com.

In our previous work, we demonstrated that preconditioning murine pancreatic KPC3 tumors with reovirus (Reo) potently sensitized these solid tumors for otherwise ineffective CD3-bsAb therapy (abbreviated to Reo&CD3-bsAbs; ref. 8). KPC3 tumors display many characteristics of human PDAC, including desmoplastic stroma containing α-smooth muscle actin (αSMA)^+^ fibroblasts and collagen, and the absence of CD8^+^ T cells (Fig. 1A). KPC3 tumors also display TGF-β signaling, as indicated by nuclear accumulation of epithelial and stromal phosphorylated Smad2, a signaling protein directly downstream of the TGF-β type I receptor. Similarly to the murine pancreatic KPC3 tumor model, murine colon MC38 tumors display phosphorylated Smad2, but they do not contain many αSMA^+^ fibroblasts and collagen and show a basal presence of CD8^+^ T cells (Fig. 1B). Because TGF-β signaling is active in both KPC3 and MC38 tumor tumors (23) and TGF-β has many immunoinhibitory characteristics, we hypothesized that inhibition of TGF-β might enhance the efficacy of Reo&CD3-bsAb therapy in these models.

First, we assessed the effect of TGF-β blockade as a monotherapy. We employed the murine mAb 1D11 (αTGF-β), which neutralizes all three isoforms of TGF-β (32). This antibody was effective in decreasing TGF-β signaling *in vitro*, as was determined using a transcriptional reporter assay (CAGA-luciferase; Supplementary Fig. S1A) and phosphorylation of Smad2 (Supplementary Fig. S1B). We next assessed the effect of TGF-β inhibition *in vivo* by applying TGF-β blockade in immunocompetent mice bearing subcutaneous KPC3 or MC38 tumors. Interestingly, TGF-β blockade significantly delayed tumor outgrowth of both KPC3 and MC38 tumors, but only when TGF-β blockade was started early after tumor challenge (Fig. 1C and D) and not when tumors were already established (Supplementary Fig. S2A). Especially in KPC3 tumors, this delay in tumor growth after early, but not late intervention with TGF-β-blocking antibodies was accompanied by a decreased intratumoral collagen deposition (Fig. 1E and F; Supplementary Fig. S2B). The impaired outgrowth of KPC3 tumors after TGF-β blockade could not be attributed to lower proliferation of tumor cells, because the frequency of Ki67^+^ cells was not affected (Fig. 1G). In addition, the same delay in KPC3 tumor growth after early TGF-β blockade could be observed in immunodeficient NSG mice that lack T, B, and natural killer (NK) cells, suggesting that this delay in tumor growth after TGF-β blockade is not immune-mediated (Fig. 1H). Combined, these data demonstrate that early TGF-β blockade delays outgrowth of both KPC3 and MC38 tumors, which could possibly lead to improved efficacy of Reo&CD3-bsAb therapy.

### TGF-β Blockade does not Impair Reo Replication and the Reo-induced Interferon Response

**FIGURE 2:**
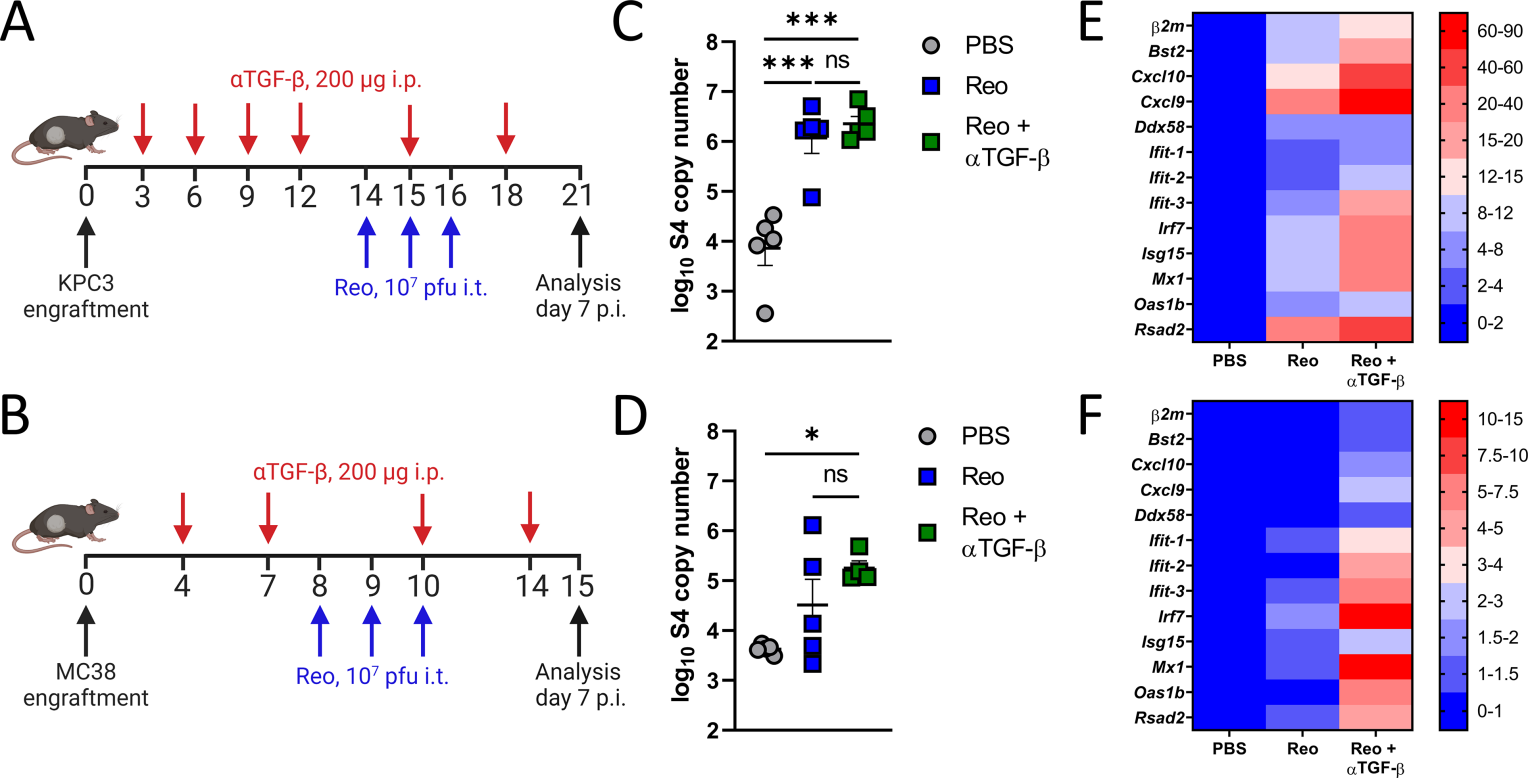
TGF-β blockade does not impair Reo replication and the Reo-induced IFN response in KPC3 and MC38 tumors. Mice (*n* = 4–5/group) were engrafted subcutaneously with KPC3 cells (1 × 10^5^/mouse; **A**) or MC38 cells (5 × 10^5^/mouse; **B**) and received TGF-β-neutralizing antibodies (αTGF-β, 200 μg/injection every 3 days) starting directly after tumor engraftment. Mice received Reo intratumorally on indicated days (10^7^ pfu/injection). Mice were sacrificed on day 21 (KPC3) or day 15 (MC38) for intratumoral analysis. Reovirus genomic segment 4 (S4) copy number in KPC3 (**C**) or MC38 (**D**) tumor lysates, as determined by qRT-PCR. Heatmap with relative expression of ISGs target genes in KPC3 (**E**) or MC38 (**F**) tumors after indicated treatments, as determined by qRT-PCR. Data represent mean ± SEM. Significance between groups in B and E was determined using an ordinary two-way ANOVA with Tukey *post hoc* test. Significance levels: ns, not significant; *, *P* < 0.05; ***, *P* < 0.001. Figures A and D were created with BioRender.com.

Before investigating the effect of TGF-β blockade on the efficacy of Reo&CD3-bsAb therapy, we first analyzed whether TGF-β blockade would not affect the replication and immune-stimulatory properties of Reo in KPC3 and MC38 tumors. *In vitro,* Reo replication was not altered in KPC3 and MC38 cells after the addition of recombinant TGF-β or TGF-β inhibition (Supplementary Fig. S3). To confirm this *in vivo*, immunocompetent mice were treated with αTGF-β or left untreated and palpable tumors were injected with Reo. Reo replication and the Reo-induced expression of IFN-stimulated genes (ISG) were compared between groups at the end of the experiment (Fig. 2A and B). In both KPC3 and MC38 tumors, Reo replication (Fig. 2C and D) and the Reo-induced expression of ISGs including T cell–attracting chemokines *Cxcl9* and *Cxcl10* (Fig. 2E and F) were not negatively affected after TGF-β blockade. Instead, the expression of various ISGs was higher in the groups that received Reo + αTGF-β compared with the group that received Reo only. Combined, these data indicate that TGF-β inhibition does not negatively influence the Reo-induced inflammatory response in the TME.

### TGF-β Blockade Enhances the Reo-induced Influx of T Cells in MC38 Tumors But not in KPC3 Tumors

**FIGURE 3:**
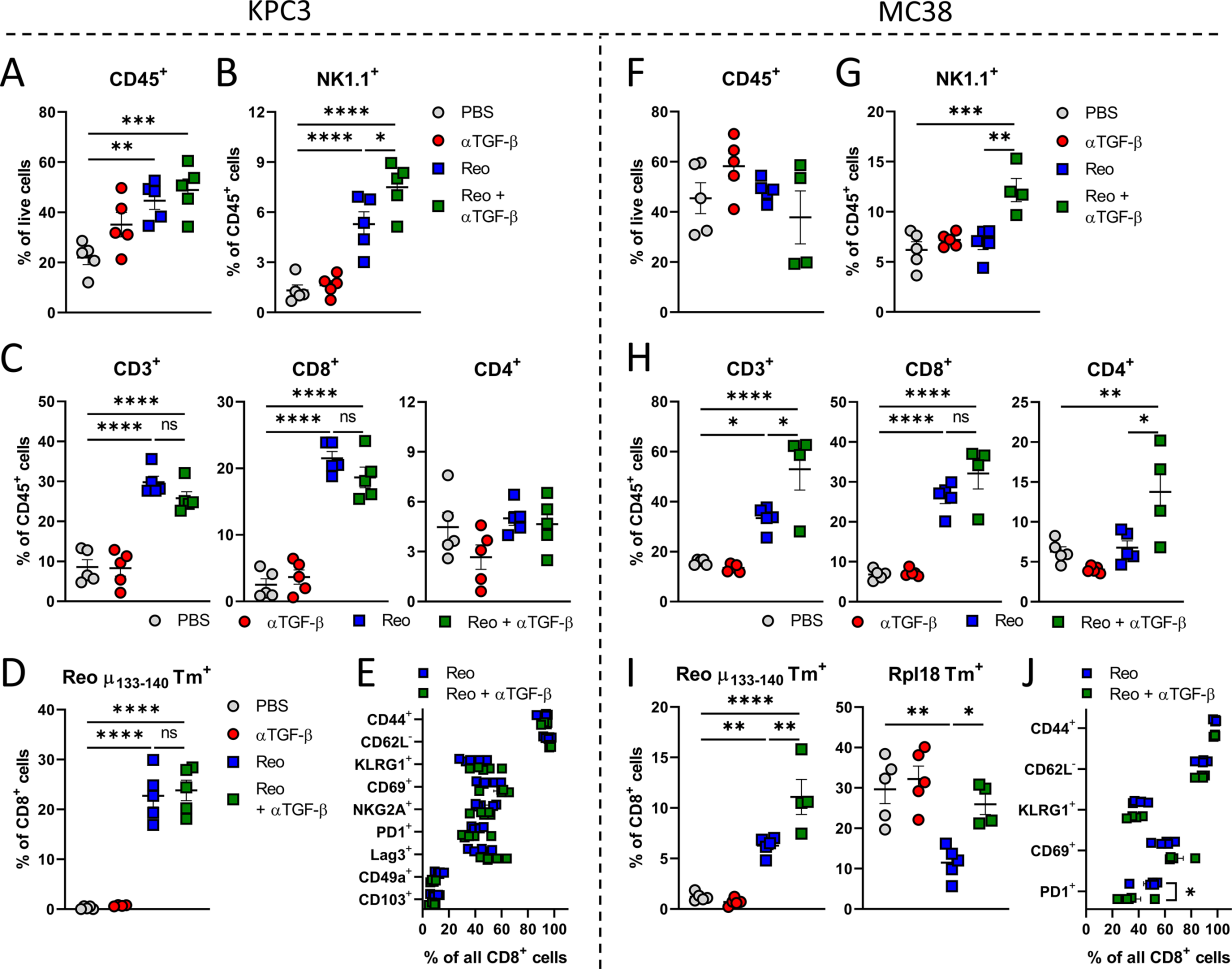
TGF-β blockade enhances the Reo-induced influx of T cells in MC38 tumors but not in KPC3 tumors. Experiments were performed according to the design described before in Fig. 2A (KPC3) and Fig. 2B (MC38). **A,** Frequency of CD45^+^ immune cells in KPC3 tumors after indicated treatments. **B,** Frequency of NK cells within the CD45^+^ immune cell population in KPC3 tumors. **C,** Percentage of CD3^+^, CD8^+^, and CD4^+^ T cells within CD45^+^ immune cells in KPC3 tumors. **D,** Frequency of reovirus-specific μ1_133–140_ T cells within the intratumoral CD8^+^ T-cell population. **E,** Expression of various markers on intratumoral CD8^+^ T cells after receiving Reo only or Reo + αTGF-β. **F,** Frequency of CD45^+^ immune cells in MC38 tumors after indicated treatments. **G,** Frequency of NK cells within the CD45^+^ immune cell population in MC38 tumors. **H,** Percentage of CD3^+^, CD8^+^, and CD4^+^ T cells within CD45^+^ immune cells in MC38 tumors. **I,** Frequency of reovirus-specific μ1_133–140_ and tumor-specific Rpl18 T cells within the intratumoral CD8^+^ T-cell population. **J,** Expression of various markers on intratumoral CD8^+^ T cells after receiving Reo only or Reo + αTGF-β. Data represent mean ± SEM. Significance in A–D and F–I was determined using an ordinary one-way ANOVA with Tukey multiple comparisons test. Significance between groups in E and J was determined using an ordinary two-way ANOVA with Tukey *post hoc* test. Significance levels: ns, not significant; *, *P* < 0.05; **, *P* < 0.01; ***, *P* < 0.001; and ****, *P* < 0.0001.

The efficacy of reovirus-based immunotherapy such as Reo&CD3-bsAb therapy relies on efficient Reo-induced intratumoral T-cell influx. Because TGF-β is known to promote an immunosuppressive and T cell–excluding environment in the TME, we hypothesized that TGF-β blockade might further enhance the Reo-induced T-cell influx and function in these tumors. In KPC3 tumors, TGF-β blockade did not enhance the influx of total CD45^+^ immune cells (Fig. 3A) but significantly increased the frequency of NK cells after Reo administration (Fig. 3B). Surprisingly, however, TGF-β blockade did not improve the Reo-induced influx of (reovirus-specific) CD8^+^ T cells, nor their activation status (Fig. 3C–E). TGF-β blockade also did not enhance total CD45^+^ immune cell influx in MC38 tumors (Fig. 3F), and again significantly improved the frequency of NK cells (Fig. 3G). Compared with KPC3 tumors, PBS-treated MC38 tumors already contained a higher basal frequency of CD8^+^ T cells (6.808 ± 0.57 vs. 2.502 ± 0.92) within the CD45^+^ immune cell population. In contrast to KPC3 tumors, αTGF-β administration significantly increased the Reo-induced influx of total T cells in MC38 tumors (Fig. 3H), as well as the frequency of reovirus-specific (μ1_133–140_ Tm^+^) and tumor-specific (Rpl18 Tm^+^) CD8^+^ T cells compared with the group that received Reo only (Fig. 3I). Expression of various activation markers on CD8^+^ T cells was again comparable between both Reo-treated groups (Fig. 3J). Combined, these data indicate that TGF-β blockade does not improve the Reo-induced T-cell influx and activation in KPC3 tumors. However, in MC38 tumors, the frequency of T cells in the tumor, including reovirus- and tumor-specific T cells, is significantly enhanced when TGF-β signaling is inhibited.

### Reovirus Administration Increases TGF-β Signaling in KPC3, but not MC38 Tumors

**FIGURE 4:**
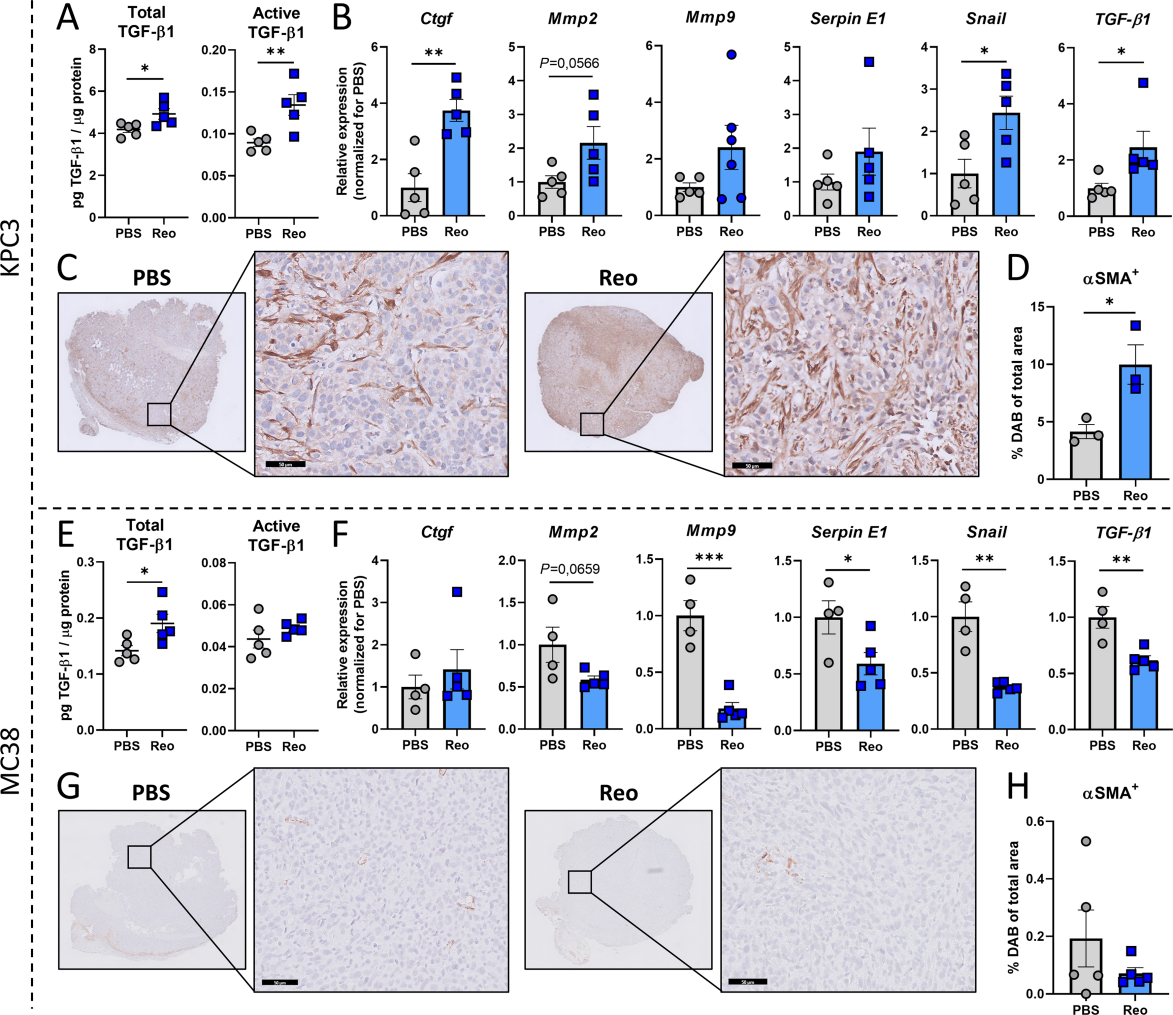
Reovirus administration increases TGF-β signaling in KPC3, but not MC38 tumors. **A,** Levels of active and total TGF-β in tumor lysates of KPC3 tumors (*n* = 4–5/group) treated intratumorally with PBS or Reo (3 × 10^7^ pfu) and harvested after 5 days. **B,** Relative expression of TGF-β target genes in PBS- or Reo-treated KPC3 tumors (*n* = 4–5/group), as determined by qRT-PCR. **C,** Representative images obtained from IHC staining of PBS- or Reo-treated KPC3 tumors (*n* = 3–5/group) for αSMA. Scale bars of magnification images equal 50 μmol/L. **D,** Quantification of positive DAB signal in sections stained for αSMA. **E,** Levels of active and total TGF-β in tumor lysates of MC38 tumors (*n* = 4–5/group) treated intratumorally with PBS or Reo (3 × 10^7^ pfu) and harvested after 5 days. **F,** Relative expression of TGF-β target genes in PBS- or Reo-treated MC38 tumors (*n* = 4–5/group), as determined by qRT-PCR. **G,** Representative images obtained from IHC staining of PBS- or Reo-treated MC38 tumors (*n* = 3–5/group) for αSMA. Scale bars of magnification images equal 50 μmol/L. **H,** Quantification of positive DAB signal in sections stained for αSMA. Data represent mean ± SEM. Significance between PBS and Reo in A, B, D, E, and H was determined using unpaired *t* tests. Significance levels: *, *P* < 0.05; **, *P* < 0.01; and ***, *P* < 0.001.

Next, we explored whether Reo administration affects TGF-β signaling in these tumors. Interestingly, when Reo was administered to mice bearing KPC3 tumors, a further increase in the presence of TGF-β1 levels in the tumor was observed (Fig. 4A). Expression of various TGF-β target genes was also elevated within the tumor lysate (Fig. 4B). Furthermore, Reo-treated tumors contained more αSMA^+^ fibroblasts (Fig. 4C and D), which are known to be induced by TGF-β (33). Together, these data suggest that TGF-β signaling is increased in KPC3 tumors after Reo administration, which provides an additional rationale to apply TGF-β blockade in combination with Reo-based viro-immunotherapy. In contrast, MC38 tumors displayed much lower total and active TGF-β1 levels in the tumor compared with KPC3 tumors, and the presence of active TGF-β was not increased upon Reo administration (Fig. 4E). In addition, expression of TGF-β target genes was decreased in Reo-treated MC38 tumors (Fig. 4F) and the intratumoral presence of αSMA-positive fibroblasts was not increased (Fig. 4G and H). We conclude that Reo differentially impacts TGF-β signaling in KPC3 and MC38 tumors, which might influence the added value of TGF-β blockade on the efficacy of Reo&CD3-bsAbs in these preclinical models.

### TGF-β Blockade Diminishes the Efficacy of Reo&CD3-bsAb Therapy in the Pancreatic KPC3.TRP1 Tumor Model

**FIGURE 5:**
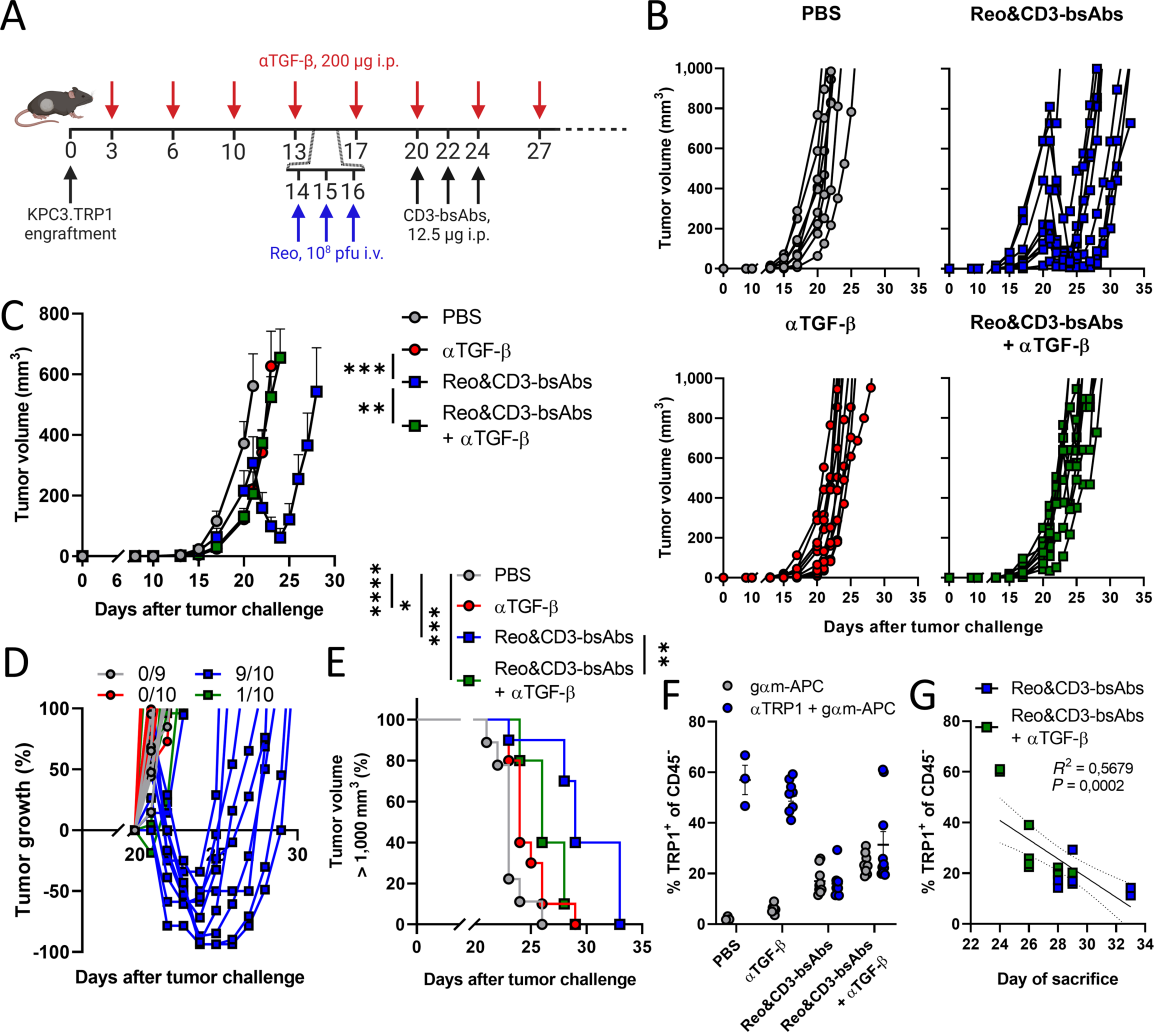
TGF-β inhibition diminishes the efficacy of Reo&CD3-bsAb therapy in the KPC3.TRP1 tumor model. **A,** Overview of the experiment described in **B–G**. Mice (*n* = 9–10/group) were subcutaneously engrafted with KPC3.TRP1 cells (1 × 10^5^/mouse) and received TGF-β-neutralizing antibodies (αTGF-β, 200 μg/injection every 3 days) starting directly after tumor engraftment. Mice received Reo intravenously on days 14, 15, and 16 (10^8^ pfu/injection) and received CD3-bsAbs intraperitoneally (12.5 μg/injection) on days 20, 22, and 24. Tumor growth was measured 3–5×/week. **B,** Individual tumor growth curves of mice receiving indicated treatments. **C,** Average tumor growth curves of mice receiving indicated treatments. One nonresponding mouse in the Reo&CD3-bsAbs group is excluded for clarity (see also B). Significant differences in average tumor growth were calculated on day 23. **D,** Relative changes in tumor volume of individual mice, calculated from the start of CD3-bsAb treatment. Indicated is the number of mice with tumor regressions in each group. **E,** Kaplan–Meier survival graphs of mice after indicated treatments. **F,** Quantification of TRP1 expression on CD45^−^ cells within the end-stage KPC3.TRP1 tumors after indicated therapies. Gray values indicate corresponding background staining of secondary goat-anti-mouse antibody only. **G,** Correlation between TRP1 expression in tumors and the day of sacrifice. Data represent mean ± SEM. Significance in C was determined using an ordinary one-way ANOVA with Tukey multiple comparisons test. Log-rank tests were used to compare differences in survival in E. Significance levels: *, *P* < 0.05; **, *P* < 0.01; ***, *P* < 0.001; and ****, *P* < 0.0001. Figure A was created with BioRender.com.

We first employed the KPC3 tumor model to test our hypothesis that TGF-β blockade could improve the antitumor efficacy of Reo&CD3-bsAbs therapy. Immunocompetent mice were engrafted with a KPC3 tumor expressing TRP1 as a model antigen to be targeted by CD3-bsAbs (Fig. 5A). As reported previously (8), Reo&CD3-bsAbs therapy induced steep regressions (Fig. 5B and C), followed by tumor escape. Unexpectedly, however, TGF-β blockade did not improve Reo&CD3-bsAb therapy but abrogated its antitumor efficacy. Tumors of mice that received Reo&CD3-bsAbs as well as TGF-β blockade did not regress in size after receiving CD3-bsAbs but displayed similar tumor growth as observed in mice treated with TGF-β blockade alone (Fig. 5C and D). Ultimately, Reo&CD3-bsAbs + αTGF-β treated-mice did have significantly better survival compared with untreated mice, but their survival was significantly worse compared with mice that received Reo&CD3-bsAbs without TGF-β inhibition (Fig. 5E).

The impaired efficacy of Reo&CD3-bsAbs, when combined with TGF-β blockade, could not be attributed to a lower presence of T cells, because tumors that received this triple combination therapy did not demonstrate lower intratumoral T-cell frequencies compared with the group that received Reo&CD3-bsAbs without αTGF-β (Supplementary Fig. S4A). Instead, there was a trend toward a higher T-cell presence in tumors after TGF-β blockade and Reo&CD3-bsAb therapy compared with the group that only received Reo&CD3-bsAb therapy, mimicking the increased T-cell influx after TGF-β blockade that was observed in MC38 tumors (Fig. 3H). Expression levels of various T-cell activation markers were also similar between both groups (Supplementary Fig. S4B). Histologic analysis confirmed that tumors of the Reo&CD3-bsAbs + αTGF-β group contained a high number of CD3^+^ T cells that were spread throughout the whole tumor (Supplementary Fig. S4C and S4D). These data indicate that TGF-β inhibition did not impair the reovirus-induced quantity or location of effector T cells in these end-stage KPC3.TRP1 tumors.

Because the impaired response to Reo&CD3-bsAb therapy after TGF-β blockade could not be attributed to a lower frequency of T cells, we next investigated whether an impaired quality of T cells might explain this effect. CD8^+^ T cells are the main effector cells that infiltrate into the tumor after reovirus administration and are employed by CD3-bsAbs (28). *In vitro* experiments showed that the CD3-bsAb–induced cytotoxic efficacy of naïve CD8^+^ T cells was not impaired when TGF-β was added or neutralized (Supplementary Fig. S5A). Similarly, T cells of CD8 TβRII KO mice that selectively lacked TGF-β signaling in their CD8^+^ T cells demonstrated similar cytotoxic capacity as TβRII WT T cells (Supplementary Fig. S5B). To confirm this *in vivo*, TβRII WT or CD8 TβRII KO mice were inoculated with KPC3.TRP1 tumor cells and received Reo&CD3-bsAb therapy as described earlier (Supplementary Fig. S5C). Interestingly, the efficacy of Reo&CD3-bsAb therapy was similar in TβRII WT and CD8 TβRII KO mice, while again Reo&CD3-bsAb + αTGF-β therapy demonstrated decreased antitumor effects and survival (Supplementary Fig. S5D and S5E).

Further flow cytometry analysis of end-stage tumors that received Reo&CD3-bsAbs as well as TGF-β blockade confirmed that TGF-β did not affect T-cell function. Tumors of mice that received Reo&CD3-bsAbs + αTGF-β demonstrated loss of TRP1 expression in the majority of CD45^−^ cells, similar to tumors of mice treated with Reo&CD3-bsAb (Fig. 5F), a phenomenon previously described in mice with successful tumor regressions upon Reo&CD3-bsAb treatment (8). Indeed, TRP1 expression in these groups negatively correlated with survival time until the experimental endpoint (Fig. 5G), indicating that the best clinical response was correlated with the highest loss of TRP1 expression. Importantly, αTGF-β alone did not decrease the number of TRP1-expressing CD45^−^ cells, indicating that the decreased frequency of TRP1-expressing cells after Reo&CD3-bsAb + αTGF-β was due to active attack and T cell–mediated killing of TRP1-expressing cells, and not because TGF-β blockade simply decreases TRP1 expression. Altogether, these data indicate TGF-β blockade impairs the efficacy of Reo&CD3-bsAb therapy in the KPC3 tumor model, even though the intratumoral T-cell frequency and their cytotoxic capacity were not negatively affected by TGF-β signaling inhibition.

### TGF-β Blockade Significantly Enhances the Efficacy of Reo&CD3-bsAb Therapy in the MC38.TRP1 Model of Colon Cancer

**FIGURE 6:**
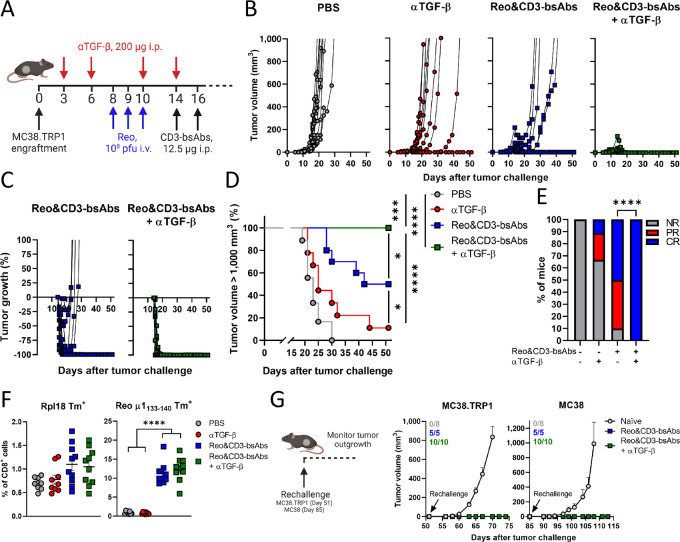
TGF-β blockade significantly enhances the efficacy of Reo&CD3-bsAb therapy in the MC38.TRP1 model of colon cancer. **A,** Overview of the experiment described in **B–H**. Mice (*n* = 9–10/group) were subcutaneously engrafted with MC38.TRP1 cells (5 × 10^5^/mouse) and received TGF-β-neutralizing antibodies (αTGF-β, 200 μg/injection every 3 days) starting directly after tumor engraftment. Mice received Reo (intravenously, 10^8^ pfu/injection) and CD3-bsAbs (intraperitoneally, 200 μg/injection) on days 14 and 16. Tumor growth was measured 3×/week. **B,** Individual tumor growth curves of mice receiving indicated treatments. **C,** Relative changes in tumor volume of individual mice from the start of CD3-bsAb treatment. **D,** Kaplan–Meier survival graphs of mice after indicated treatments. **E,** Frequency of nonresponders (NR), partial responders (PR; tumor regression/stagnation for more than 7 days), or complete responders (CR) within indicated treatment groups. **F,** Frequency of Rpl18^+^ and Reo μ1_133–140_ CD8^+^ T cells in the blood of mice after indicated treatments. **G,** Rechallenge experiment. All CR mice from D were subcutaneously engrafted with MC38.(TRP1) tumor cells (5 × 10^5^/mouse) in the alternate flank on day 51 (MC38.TRP1) or day 85 (MC38) and tumor outgrowth was measured 3×/week. Indicated is the number of mice within each group that rejected the rechallenge. Data represent mean ± SEM. Log-rank tests were used to compare differences in survival in D. A *χ*^2^ test was used to determine statistical differences in response in E. Significance between groups in F was determined using an ordinary one-way ANOVA with Tukey multiple comparisons test. Significance levels: *, *P* < 0.05; **, *P* < 0.01; ***, *P* < 0.001; and ****, *P* < 0.0001. Figures A and G were created with BioRender.com.

We next investigated whether TGF-β blockade could improve the efficacy of Reo&CD3-bsAb therapy in the MC38 tumor model, which also displays high TGF-β signaling. Because MC38 tumor cells do not naturally express tumor antigen TRP1, we transfected MC38 cells with a plasmid encoding TRP1 and sorted TRP1^+^ cells (Supplementary Fig. S6A and S6B), similar to what was previously done for KPC3. Hereafter, MC38.TRP1 cells were susceptible to T cell–mediated killing in the presence of CD3-bsAbs in an *in vitro* setting (Supplementary Fig. S6C), so we continued investigating whether TGF-β inhibition would improve the antitumor efficacy Reo&CD3-bsAb therapy in mice bearing MC38.TRP1 tumors (Fig. 6A). TGF-β blockade alone already delayed the outgrowth of MC38 tumors and induced complete tumor clearance in 1 of 9 animals ( = 11.1%; Fig. 6B). In this model, Reo&CD3-bsAb therapy led to durable responses with complete tumor clearance in 50% of the animals (Fig. 6B). Most interestingly, however, was the observation that here the efficacy of Reo&CD3-bsAb therapy was significantly improved by TGF-β inhibition. TGF-β inhibition combined with Reo&CD3-bsAb therapy led to very rapid tumor clearance in 100% of animals and significantly enhanced survival (Fig. 6C–E). This increase in therapeutic efficacy could not be attributed to an increased presence of tumor-specific (Rpl18 Tm^+^) or reovirus-specific (μ1_133–140_ Tm^+^) CD8^+^ T cells in the circulation, because their frequencies were similar between the group that received Reo&CD3-bsAb therapy and the group that received additional αTGF-β therapy (Fig. 6F).

Because 50% of mice that received Reo&CD3-bsAb therapy and 100% of mice that received Reo&CD3-bsAb therapy in combination with αTGF-β completely cleared their tumor, we wondered whether tumor-specific immunologic memory was established. All mice that cleared the first tumor received a rechallenge at the alternate flank with MC38.TRP1 tumor cells, which was rejected (Fig. 6G). Similarly, a third rechallenge with the parental MC38 cell line was also rejected, suggesting the establishment of an effective antitumor memory immune response. Combined, these data indicate that Reo&CD3-bsAb therapy alone is already effective in clearing MC38 tumors and establishing antitumor immunity, but the addition of αTGF-β significantly increases the primary antitumor response.

Altogether, we demonstrated that the addition of TGF-β blockade has the potential to improve the efficacy of Reo&CD3-bsAb therapy, but this benefit depends on the tumor model used. Although both KPC3 and MC38 tumors display active TGF-β signaling, the therapeutic efficacy of Reo&CD3-bsAbs was only drastically improved when TGF-β signaling was inhibited in MC38 tumors and not in KPC3 tumors. This differential effect of TGF-β blockade during Reo&CD3-bsAb combination therapy was associated with a different effect of Reo on TGF-β signaling in these tumors. Further understanding of intertumor differences that might contribute to this differential effect of TGF-β blockade is essential to improve, and not impair, the efficacy of viroimmunotherapeutic strategies.

## Discussion

In this study, we demonstrated that the combination therapy of reovirus and CD3-bispecific antibodies (Reo&CD3-bsAb) can be significantly improved by additional neutralization of TGF-β. However, the added benefit of TGF-β blockade is model dependent. Our data indicate that inhibition of TGF-β signaling might be a promising strategy to enhance the efficacy of viroimmunotherapeutic strategies, but intertumor differences might also result in the diminishing of their efficacy after TGF-β blockade.

TGF-β is mostly recognized as a tumor-promoting cytokine by inducing cancer cell migration and invasion (34, 35) and as an immunosuppressive factor by inhibiting the generation and effector function of CD4^+^ and CD8^+^ effector T cells (17). The tumor-promoting and immunoinhibitory characteristics of TGF-β make it an attractive target for therapeutic intervention to enhance the efficacy of (viro-)immunotherapeutic strategies.

In preclinical research, 1D11 is a well-known antibody that prevents the binding of TGF-β isoforms to TGF-β receptors (32). TGF-β blockade using 1D11 only induced suppression of tumor growth when TGF-β blockade was initiated directly after tumor challenge (early intervention), and not when αTGF-β treatment was initiated when tumors were already established (late intervention). Similar observations were made in a MDA-MB-231 model of bone metastasis, where the reduced tumor burden in the bones after TGF-β inhibition was much more pronounced when TGF-β blockade was administered directly after tumor inoculation, compared with administration when metastases in the bones were already established (36). In addition, treatment of established, orthotopic MDA-MB-231 tumors with 1D11 did not impact tumor growth, while stable overexpression of a soluble TGF-βRII (i.e., continued neutralization of TGF-β) almost completely blocked the growth of the same tumor (37). For KPC3 tumors, the impaired tumor growth suppression after early TGF-β blockade was not immune mediated and could not be associated with impaired proliferation, but was associated with decreased intratumoral collagen disposition, as has also been observed in the murine mammary carcinoma 4T1 model and the human mammary carcinoma MDA-MB-231 model (37, 38). These combined observations suggest that the TGF-β blockade–induced delay in tumor growth might be a result of microenvironmental changes, rather than a direct effect on tumor cells.

In our studies, we observed that TGF-β inhibition using 1D11 did not improve the efficacy of Reo&CD3-bsAb therapy in the murine pancreatic KPC3 model, but did significantly enhance the number of responders and overall survival in the murine colon MC38 model. A similar contrast was observed in a study where TGF-β inhibition enhanced the efficacy of checkpoint blockade in the MC38 tumor model but was unable to do so in a model for murine pancreatic cancer (23). The divergent effects of TGF-β blockade have also been observed in a panel of 12 models for metastatic breast cancer, where TGF-β using 1D11 suppressed the formation of lung metastasis in 42% of the models, did not induce a response in 33% of the models and induced an increase in lung metastasis in 25% of the models (39). An understanding of the factors underlying this dichotomy would be a first step toward predicting which individuals would most likely benefit from TGF-β neutralization in addition to viro-immunotherapy.

First, we took a closer look at the composition of the TME in both tumors. One big difference between the tumor models used is the immunogenicity and the related baseline frequency of tumor-infiltrated immune cells. The chemically-induced MC38 tumor model is more immunogenic compared with the genetically-induced KPC3 tumor model. Higher immunogenicity is associated with higher therapeutic efficacy of TGF-β inhibition, as was observed in a study where TGF-β inhibition using kinase inhibitor galunisertib resulted in stronger CD8^+^ T-cell dependent control of tumor growth of immunogenic 4T1-luciferase breast tumors, compared with poorly immunogenic 4T1 parental tumors (40). Similarly, TGF-β blockade in multiple squamous cell carcinoma (SCC) models using the pan-TGF-β neutralizing antibody was most effective in SCC tumors with highest mutational loads (19). Immunogenic MC38 tumors already contain more T cells at baseline compared with poorly immunogenic KPC3 tumors, and TGF-β inhibition was able to further enhance the reovirus-induced influx of T cells in MC38 tumors. Interestingly, previous studies indicated that the main mechanism of action of TGF-β blockade to improve the efficacy of checkpoint blockade is by increasing T-cell infiltration into the tumor (21, 41). Our data suggest that this might also be valid for other immunotherapeutic strategies, including Reo&CD3-bsAb therapy.

Another difference between the TME of both tumor models is the abundance of stroma in KPC3 tumors, which is absent in MC38 tumors. The tumor stroma consists, among other components, of fibroblasts, matrix proteins, and the vasculature (42). The importance of tumor stroma for the development, promotion, and invasion of cancer has become increasingly clear. In particular, cancer-associated fibroblasts can stimulate the growth, invasion, angiogenesis, and metastasis of tumors (43). As such, various stroma-related factors, such as an abundance of αSMA^+^ fibroblasts and high expression of fibroblast activation protein (FAP), are associated with aggressive disease progression, recurrence, and therapy resistance in pancreatic and colorectal cancer (44–47). Matrix proteins such as type I collagens can promote the proliferation and invasiveness of tumor cells (48, 49). High collagen content and cross-linking also contribute to tumor stiffness and drive metastatic growth (50). Interestingly, collagen can also decrease responses to immunotherapy by acting as a physical barrier to immune cell infiltration, as well as delivering inhibitory signals to immune cells such as T and NK cells by binding to the leukocyte-associated immunoglobulin-like receptor-1 (LAIR-1; ref. 51). Although TGF-β inhibition was able to decrease αSMA^+^ fibroblast and collagen content in KPC3 tumors, this decrease might not have been sufficient to enhance the efficacy of Reo&CD3-bsAb therapy similarly as was observed in MC38 tumors where the stromal compartment is mostly absent.

In addition, besides the difference in T-cell infiltration or stromal composition, tumor-intrinsic differences might explain the differential effects of TGF-β inhibition on therapy outcome. Both KPC3 and MC38 tumor models used in this study display active signaling of TGF-β. Canonical TGF-β signaling involves the formation of a heterooligomer complex comprising Smad4 and other Smad proteins, that travels to the nucleus to induce expression of TGF-β target genes (52). Alternatively, TGF-β signaling can also occur noncanonically, in a Smad4-independent manner. While canonical TGF-β signaling is involved in both tumor-promoting and tumor-suppressive pathways, noncanonical TGF-β signaling especially activates tumor-promoting pathways that facilitate EMT and cell migration, such as the RAS/RAF/MEK/ERK pathway. Interestingly, unlike KPC3, MC38 cells do not display Smad4-dependent signaling, even though Smad2 is phosphorylated (53). This lack of Smad4 expression results in enhanced tumorigenicity and metastatic potential, which could be reduced when Smad4 was introduced in these cells (53). Thus, Smad4 loss might result in the uncoupling of the TGF-β-mediated growth-suppressive function from its pro-oncogenic effects (54), which might explain why especially in the MC38 model TGF-β inhibition was very effective. Indeed, ablation of Smad4 expression in murine pancreatic 6694c2 tumors enhanced T-cell influx and improved the response to chemoimmunotherapy (55). Because both canonical and noncanonical TGF-β signaling pathways are intact in the KPC3 model, TGF-β inhibition might not only lead to the inhibition of its tumor-promoting pathways but also some of its tumor-suppressive aspects. This is eloquently demonstrated in the murine pancreatic BMFA3 tumor model, where treatment with an anti-TGF-βR2 antibody significantly slowed the growth of *Tgfbr2-*mutant tumors but increased the growth of *Tgfbr*^wt^ tumors (56).

Another difference that we found between both models was the contrasting effect of Reo on TGF-β signaling. We observed that Reo administration leads to a further elevated presence of TGF-β in KPC3 tumors, which was accompanied by an increased expression of various TGF-β target genes and αSMA^+^ fibroblasts. An increase in TGF-β production after Reo administration has also been observed in other tumor models, as well as after other OV infections (57–60). In contrast, Reo administration led to decreased TGF-β signaling in MC38 tumors. This may imply that in KPC3 tumors blockade of TGF-β signaling is overruled by reovirus administration, while in MC38 tumors TGF-β blockade works synergistically with the Reo-induced decrease in TGF-β signaling and thereby results in significantly improved antitumor responses in these tumors. However, these opposite effects of Reo administration on TGF-β production and the expression of TGF-β target genes may not necessarily involve the canonical TGF-β signaling pathway, because MC38 tumor cells lack Smad4-mediated responses and the expression of many TGF-β target genes can also be induced or inhibited by other pathways.

In conclusion, we demonstrated that TGF-β blockade can differentially affect the efficacy of Reo&CD3-bsAb therapy in different preclinical tumor models, even if both models display active TGF-β signaling at baseline. These opposite effects might be attributed to the baseline T-cell density, immunogenicity, stromal composition, genetic factors including Smad4 deficiency, the effect of TGF-β blockade on the reovirus-induced T-cell influx into the tumor, or the effect of reovirus administration on TGF-β signaling. Further understanding of these intermodel differences that dictate whether TGF-β blockade promotes or impairs viro-immunotherapy is needed to guide further therapeutic developments. Because both oncolytic virus-based immunotherapeutic strategies (61), as well as several therapeutic approaches to inhibit TGF-β signaling (52), are in clinical development, the implications of this research may be valuable for clinical practice.

## Supplementary Material

Supplementary Methods SM1 Supplementary Methods 1. Methods for immunohistochemistry, Western Blotting, TGF-β1 ELISA and in vitro assays.

Table TS1 Table S1. List of antibodies used for flow cytometric analysis.

Table TS2 Table S2. List of primers used for RT-qPCR analysis.

Figure S1 Figure S1. Inhibition of TGF-β signaling by the monoclonal antibody 1D11.

Figure S2 Figure S2. Late TGF-β blockade does not affect tumor outgrowth.

Figure S3 Figure S3. TGF-β addition or blockade does not affect reovirus replication in KPC3 and MC38 cells in vitro.

Figure S4 Figure S4. TGF-β blockade does not impair Reo&CD3-bsAb efficacy by decreasing T-cell influx or activation.

Figure S5 Figure S5. CD8-specific TGF-β blockade does not impair the efficacy of Reo&CD3-bsAb therapy.

Figure S6 Figure S6. Introduction of TRP1 expression on MC38.TRP1 cells allows killing via CD3-bsAbs.
