# Supplementary material for: Intertumoral Differences Dictate the Outcome of TGF-β Blockade on the Efficacy of Viro-Immunotherapy
Source: Cancer Res Commun. 2023 Feb 23;3(2):325–37. doi: 10.1158/2767-9764.CRC-23-0019 (PMC9973387; doi:10.1158/2767-9764.CRC-23-0019)
Supplement: Supplementary Methods SM1 — Supplementary Methods 1. Methods for immunohistochemistry, Western Blotting, TGF-β1 ELISA and in vitro assays. [file crc-23-0019-s01.pdf]

Intertumoral differences dictate the outcome of TGF-β blockade on the efficacy of viro-immunotherapy

Authors: Christianne Groeneveldt, Jurriaan Q. van Ginkel, Priscilla Kinderman, Marjolein Sluijter, Lisa Griffioen, Camilla Labrie, Diana J. M. van den Wollenberg, Rob C. Hoeben, Sjoerd H. van der Burg, Peter ten Dijke, Lukas J. A. C. Hawinkels, Thorbald van Hall, Nadine van Montfoort

Supplementary Methods 1

Immunohistochemistry

Formalin-fixed tumor pieces were embedded in paraffin and then sectioned randomly at 4 µm and placed on Superfrost® Plus slides (VWR). Sections were dried overnight at 37 °C and stored at 4 °C until staining. Slides were deparaffinized and endogenous peroxidase was blocked with 0,3% hydrogen peroxidase (VWR) in methanol for 20 minutes. After rehydration, antigen retrieval was performed by boiling slides for 10 minutes in 0,01M sodium citrate (pH 6) (Merck). Non-specific binding was blocked using SuperBlock™ (ThermoFisher Scientific) before overnight incubation in PBS/1% BSA in a humified box at 4 °C or RT with rabbit anti-mouse CD3ε (clone D7A6E™, 1:200; Cell Signaling Technology), rat anti-mouse CD8a (clone 4SM15, 1:1600; eBioscience™), mouse anti-mouse α-smooth muscle actin (clone 1A4/ASM-1, 1:1600/1:3200; Progen), rabbit anti-mouse phosphorylated-Smad2 (clone 138D4, 1:50; Cell Signaling Technology), rabbit anti-mouse vimentin (clone D21H3, 1:400; Cell Signaling Technology), mouse anti-mouse pan-cytokeratin (clone PCK-26, 1:400; Sigma-Aldrich) or rabbit anti-mouse Ki67 (clone SP6, 1:300, Abcam). Hereafter, samples were incubated for 30 min at RT with biotinylated goat anti-rabbit, rabbit anti-rat, or goat anti-mouse secondary antibodies (1:200; Agilent), followed by incubation with avidin-biotin complex (VECTASTAIN® Elite® ABC HRP Kit; Vector Laboratories). Peroxidase activity was detected using the 2-component liquid DAB+ system (Agilent) according to the manufacturer's instructions for 5 min. Slides were counterstained in hematoxylin (Sigma Aldrich) for 15 seconds, dehydrated, and mounted using Entellan (Sigma Aldrich). Control sections were processed in parallel, but without incubation with primary antibody. No labeling was observed in the control sections. Collagen was stained by incubating rehydrated slides in 0.1% Sirius Red (Direct Red 80; Sigma-Aldrich) in 1.3% picric acid (Sigma-Aldrich) for 90 minutes after which slides were washed, dehydrated and mounted as described above. αSMA, CD3 and collagen immunohistochemistry stainings were quantified by measuring the positive DAB or Sirius Red signal using ImageJ and researchers analyzing the tissues were blinded to treatment groups (1).

Western Blotting

Phosphorylation of the downstream TGF-β signaling molecule Smad2 (pSmad2) in KPC3 tumor cells was analyzed by western blot as described before (2). Briefly, KPC3 cells were lysed in radioimmunoprecipitation assay (RIPA) buffer containing protease and phosphatase inhibitors using a stainless bead and the TissueLyser LT (Qiagen). Proteins (30 µg) were separated on a 10% SDS−polyacrylamide gel under reducing conditions and then transferred to a 0.45 µM PVDF membrane (Merck). After blocking for 1h at RT with 5% milk powder (Campina) in Tris-HCl-buffered saline containing 0.05% Tween-20 (TBS-T; Merck, Darmstadt, Germany), the membrane was incubated overnight at 4°C with anti-pSmad2 (Ser465/467) (clone 138D4; Cell Signaling Technology, 1:1000) or anti-β-actin (clone C4; Santa Cruz, 1:5000), followed HRP-conjugated goat anti-rabbit or anti-mouse IgG (Agilent, 1:5000) at RT for 90 minutes. After washing, proteins were detected on the Chemidoc imaging XRS+ system (Bio-Rad) using the Clarity Western ECL Substrate kit (Bio-Rad).

TGF-β1 ELISA

Snap-frozen KPC3 or MC38 tumor pieces were lysed in radioimmunoprecipitation assay (RIPA) buffer containing protease and phosphatase inhibitors using a stain-less bead and the TissueLyser LT (Qiagen). Homogenate was centrifuged at $13x10^3$ rpm for 15 minutes at 4 °C, after which supernatants were collected and stored at −80 °C until further analysis. Active and total mTGF-β1 levels were measured by using a Mouse TGF-β1 duoset ELISA kit according to the manufacturer's instructions (R&D Systems, Minneapolis, MN, USA). Absorbance was measured using the SpectraMax iD3 multi-mode plate reader (Molecular Devices). Final values were expressed per µg protein in the tumor lysate.

CAGA-Luciferase Reporter Assay

HepG2 (RRID:CVCL_0027) is a cell line derived from an human hepatoblastoma and was obtained from ATCC (HB-8065™). $1x10^6$ HepG2 cells per well were plated into a 6-wells plate. The next days,

cells were transfected with 2 μg of TGF-β/Smad inducible $(CAGA)_{12}$ luciferase transcriptional reporter construct, which encodes 12 repeats of the AGCCAGACA sequence (identified as a SMAD3/SMAD4-binding element in the human *SERPIN 1* promoter [39]) using lipofectamine 2000 transfection reagent (1:5; ThermoFisher Scientific). After overnight incubation, cells were harvested and 20.000 cells/well were plated in a 96-wells plate. After attachment, HepG2 cells were serum-starved overnight. The next day, serum free media were removed and replaced by medium containing TGF-β1 (0.001 - 5 ng/mL, Peprotech). In other wells, TGF-β1 was added in a concentration of 5 ng/mL in combination with increasing concentrations of the monoclonal TGFβ-blocking antibody (αTGF-β) 0.01 – 10 ng/mL, BioXCell). After overnight incubation, luciferase signal was measured using the Luciferase Assay System (Promega) according to manufacturer's instructions using the SpectraMax iD3 multi-mode plate reader (Molecular Devices).

Lactate dehydrogenase (LDH) assay

The ability of T cells to induce killing of tumor cells was evaluated using a colorimetric method for quantifying cellular cytotoxicity. In short, KPC3.TRP1, MC38 or MC38.TRP1 cells were irradiated at 8000 RAD and plated at a concentration of 30.000 cells/well. Splenocytes and lymph nodes were isolated from either treatment-naïve C57BL/6J, CD8 TGF-βRII KO or TGF-βRII WT mice and were enriched for CD8 T cells using the Mouse CD8 T Lymphocyte Enrichment Set – DM (BD Biosciences) or via nylon wool processing. Effector cells were added to tumor cells in an E/T ratio of 10:1 and CD3-bsAbs were added in a concentration of 1 µg/mL. In the experiment with naïve splenocytes from C57BL/6J mice, αTGF-β (100 or 10 μg/mL) were added as well. After 48 hours of incubation, 20 µL of Triton-X100 was added to wells containing tumor cells alone for 30 minutes to serve as a positive control. Hereafter, 50 µL of supernatant was harvested of all conditions and incubated for 30 minutes with 50 µL of lactate dehydrogenase reaction mix (Pierce LDH Cytotoxicity Assay Kit, ThermoFisher Scientific). Absorbance was measured at 490 using a SpectraMax iD3 multi-mode plate reader

(Molecular Devices). The percentage of cytotoxicity was calculated using the positive control as 100 % cytotoxicity. All conditions were performed in triplicate.

References

1. Schneider CA, Rasband WS, Eliceiri KW. NIH Image to ImageJ: 25 years of image analysis. Nature Methods **2012**;9:671-5
2. Hawinkels LJ, Paauwe M, Verspaget HW, Wiercinska E, van der Zon JM, van der Ploeg K*, et al.* Interaction with colon cancer cells hyperactivates TGF-β signaling in cancer-associated fibroblasts. Oncogene **2014**;33:97-107
