# Supplementary material for: Intertumoral Differences Dictate the Outcome of TGF-β Blockade on the Efficacy of Viro-Immunotherapy
Source: Cancer Res Commun. 2023 Feb 23;3(2):325–37. doi: 10.1158/2767-9764.CRC-23-0019 (PMC9973387; doi:10.1158/2767-9764.CRC-23-0019)
Supplement: Table TS1 — Table S1. List of antibodies used for flow cytometric analysis. [file crc-23-0019-s02.pdf]

Table S1. List of antibodies used for flow cytometric analysis.

Marker	Clone	Fluorochrome	Supplier
**CD45.2**	104	FITC	eBioscience
**CD3**	145-2C11	PE-CF594	BD Biosciences
**CD8α**	53-6.7	Alexa Fluor 700	eBioscience
**CD4**	RM4-5	APC	BioLegend
**Reo μ1$_{133\text{-}140}$ Tetramer**		APC	In-house
**Rpl18 Tetramer**		PE	In-house
**CD44**	IM-7	BV785	BioLegend
**CD62L**	MEL-14	BV421	BioLegend
**PD1**	29F.1A12	APC-Cy7	BioLegend
**Tim-3**	RMT3-23	PE	BioLegend
**NKG2A**	16A11	PE	eBioscience
**KLRG-1**	2F1	PE-Cy7	eBioscience
**CD69**	H1.2F3	BV605	BioLegend
**Lag3**	C9B7W	PE-Cy7	Invitrogen
**CD49a**	Ha31/8	BV786	BD Biosciences
**CD103**	2E7	BV711	BioLegend
**Ki67**	B56	BV711	BD Biosciences
**GzmB**	NGZB	PE-Cy7	BioLegend
