# Supplementary material for: Intertumoral Differences Dictate the Outcome of TGF-β Blockade on the Efficacy of Viro-Immunotherapy
Source: Cancer Res Commun. 2023 Feb 23;3(2):325–37. doi: 10.1158/2767-9764.CRC-23-0019 (PMC9973387; doi:10.1158/2767-9764.CRC-23-0019)
Supplement: Table TS2 — Table S2. List of primers used for RT-qPCR analysis. [file crc-23-0019-s03.pdf]

Table S2. List of primers used for RT-qPCR analysis.

Gene	Forward	Reverse
S4Q	5'-CGCTTTTGAAGGTCGTGTATCA-3'	5'-CTGGCTGTGCTGAGATTGTTTT-3'
Ifit-1	5'-CTGGACAAGGTGGAGAAGGT-3'	5'-AGGGTTTTCTGGCTCCACTT-3'
Ifit-2	5'-TGCTCTTGACTGTGAGGAGG-3'	5'-ATCCAGACGGTAGTTCGCAA-3'
Ifit-3	5'-GTGCAACCAGGTCGAACATT-3'	5'- AGGTGACCAGTCGACGAATT-3'
Irf7	5'-GACCGTGTTTACGAGGAACC-3'	5'-GCTGTACAGGAACACGCATC-3'
Isg15	5'-GGAACGAAAGGGGCCACAGCA-3'	5'-CCTCCATGGGCCTTCCCTCGA-3'
Oas1b	5'-AGCATGAGAGACGTTGTGGA-3'	5'-GCGTAGAATTGTTGGTTAGGCT-3'
Ddx58	5'-AAGGCCACAGTTGATCCAAA-3'	5'-TTGGCCAGTTTTCCTTGTCG-3'
Cxcl9	5'-TGGAGTTCGAGGAACCCTAGT-3'	5'-AGGCAGGTTTGATCTCCGTT-3'
Cxcl10	5'-ACGAACTTAACCACCATCT-3'	5'-TAAACTTTAACTACCCATTGATACATA-3'
Mx1	5'-GATGGTCCAAACTGCCTTCG-3'	5'-TTGTAAACCTGGTCCTGGCA-3'
β2m	5'-CTCGGTGACCCTGGTCTTT-3'	5'-CCGTTCTTCAGCATTTGGAT-3'
Bst2	5'-ACATGGCGCCCTCTTTCTATCACT-3'	5'-TGACGGCGAAGTAGATTGTCAGGA-3'
Rsad2	5'-GGTGCCTGAATCTAACCAGAAG-3'	5'-CCACGCCAACATCCAGAATA-3'
Ctgf	5'-GGCCTCTTCTGCGATTTCG-3'	5'-CCATCTTTGGCAGTGCACACT-3'
Id-1	5'-ACCCTGAACGGCGAGATCA-3'	5'-TCGTCGGCTGGAACACAT-3'
Mmp2	5'-TTCTGTCCCGAGACCGCTAT-3'	5'-GTGTAGATCGGGGCCATCAG-3'
Serpin E1	5'-GCCAACAAGAGCCAATCACA-3'	5'-AGGCAAGCAAGGGCTGAAG-3'
Snail	5'-AGCCCAACTATAGCGAGCTG-3'	5'-CCAGGAGAGAGTCCCAGATG-3'
TGF-β1	5'-CAACAATTCCTGGCGTTACC-3'	5'-TGCTGTCACAAGAGCAGTGA-3'
TGF-β2	5'-TCCCCTCCGAAAATGCCATC-3'	5'-TGCTATCGATGTAGCGCTGG-3'
TGF-β3	5'-CGACCGGATGAGCACATAGC-3'	5'-TTTGCTTCTTGAGACGCCCC-3'
Mzt2	5'-TCGGTGCCCATATCTCTGTC-3'	5'-CTGCTTCGGGAGTTGCTTTT-3'
Ptp4a2	5'-AGCCCCTGTGGAGATCTCTT-3'	5'-AGCATCACAAACTCGAACCA-3'
