# Supplementary material for: Intertumoral Differences Dictate the Outcome of TGF-β Blockade on the Efficacy of Viro-Immunotherapy
Source: Cancer Res Commun. 2023 Feb 23;3(2):325–37. doi: 10.1158/2767-9764.CRC-23-0019 (PMC9973387; doi:10.1158/2767-9764.CRC-23-0019)
Supplement: Figure S1 — Inhibition of TGF-β signaling by the monoclonal antibody 1D11. [file crc-23-0019-s04.pdf]

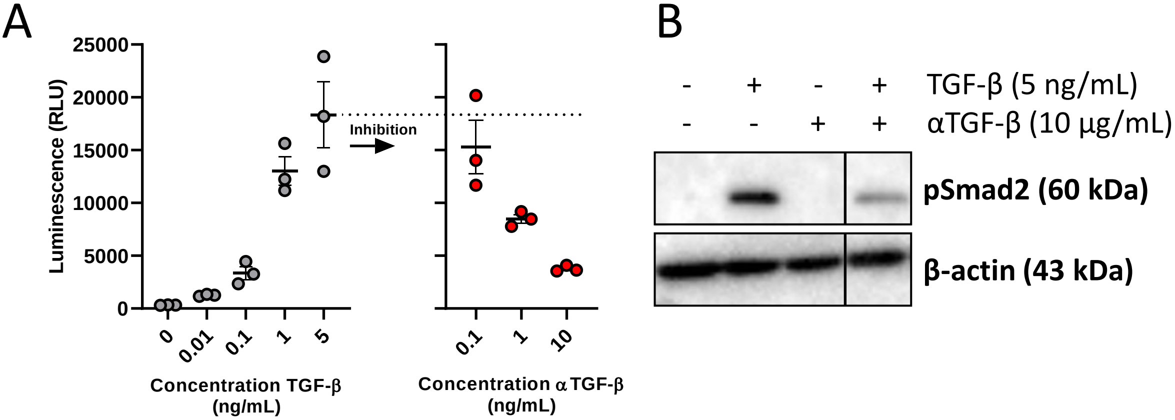

Figure S1. Inhibition of TGF-β signaling by the monoclonal antibody 1D11. (A) Induction of TGF-β signaling by TGF-β and subsequent inhibition of TGF-β signaling via TGF-β neutralizing antibodies (αTGF-β, 1D11), as measured by transcriptional CAGA-Luciferase reporter assay. Cells were incubated with TGF-β (0-5 ng/mL). In other wells with 5 ng/mL of TGF-β, αTGF-β was added (0.1-10 ng/mL). **(B)** Immunoblotting of phospho-Smad2 in KPC3 tumor cell line after TGF-β (5 ng/mL) and/or αTGF-β treatment (10 µg/mL). B-actin was measured as loading control. Vertical black line indicates cutting of blot to eliminate irrelevant samples. Data represent mean±SEM.
