# Supplementary material for: Intertumoral Differences Dictate the Outcome of TGF-β Blockade on the Efficacy of Viro-Immunotherapy
Source: Cancer Res Commun. 2023 Feb 23;3(2):325–37. doi: 10.1158/2767-9764.CRC-23-0019 (PMC9973387; doi:10.1158/2767-9764.CRC-23-0019)
Supplement: Figure S2 — Late TGF-β blockade does not affect tumor outgrowth. [file crc-23-0019-s05.pdf]

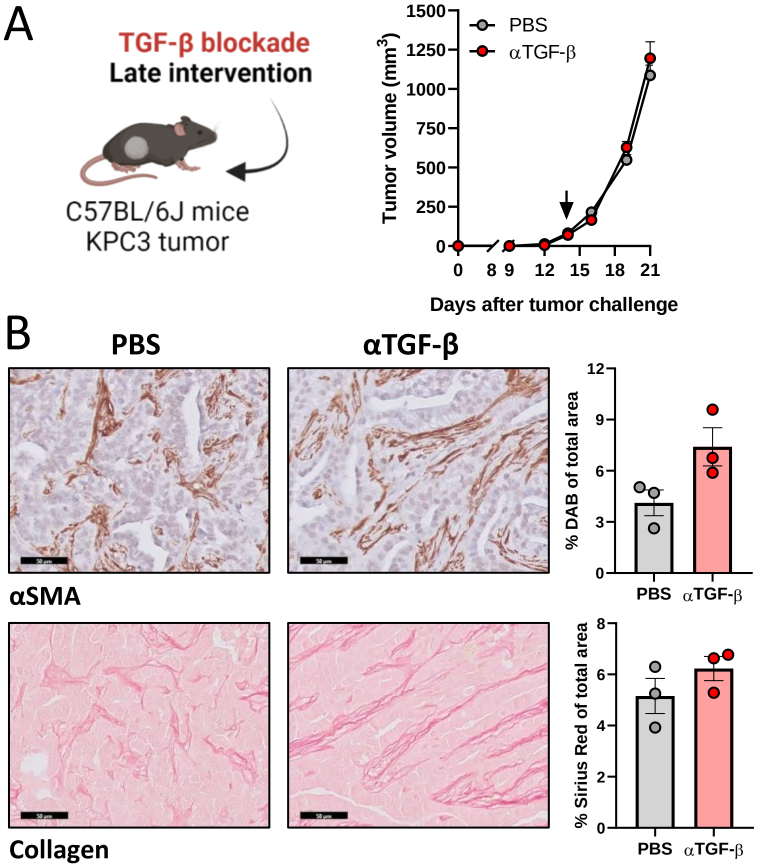

Figure S2. Late TGF-β blockade does not affect tumor outgrowth. (A) Average tumor growth curves of immunocompetent C57BL/6J mice (n=5/group) engrafted with KPC3 tumors ($1x10^5$ cells/mouse) and receiving αTGF-β (200 μg/injection every 3 days, starting on day 14, indicated by black arrow) as late intervention. **(B)** Immunohistochemistry stainings for αSMA and collagen in representative tumors after indicated treatments. Scale bars represent 50 μm and stainings were quantified using ImageJ. Data represent mean±SEM. Significance between PBS and αTGF-β was determined using unpaired t-tests. Figure (A) was created with BioRender.com.
