# Supplementary material for: Intertumoral Differences Dictate the Outcome of TGF-β Blockade on the Efficacy of Viro-Immunotherapy
Source: Cancer Res Commun. 2023 Feb 23;3(2):325–37. doi: 10.1158/2767-9764.CRC-23-0019 (PMC9973387; doi:10.1158/2767-9764.CRC-23-0019)
Supplement: Figure S3 — TGF-β addition or blockade does not affect reovirus replication in KPC3 and MC38 cells in vitro. [file crc-23-0019-s06.pdf]

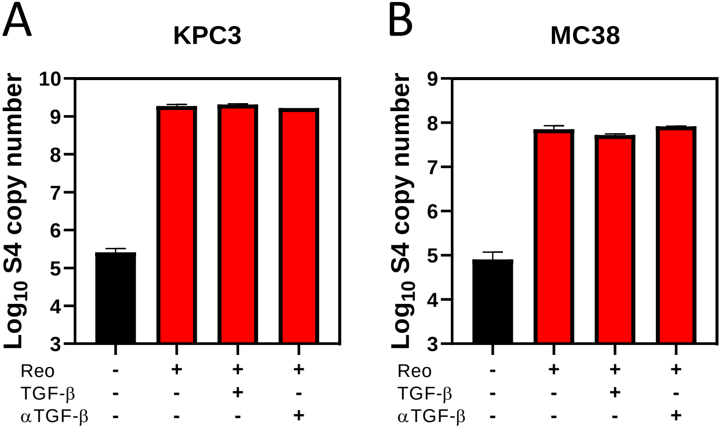

Figure S3. TGF-β addition or blockade does not affect reovirus replication in KPC3 and MC38 cells in vitro. Reovirus genomic segment 4 (S4) copy number in KPC3 **(A)** or MC38 **(B)** lysates, as determined by RT-qPCR. Cells were infected with reovirus for 24 hours (multiplicity of infection of 10) in the presence of TGF-β (5 ng/mL) or αTGF-β (10 μg/mL). Data represent mean±SEM.
