# Supplementary material for: Intertumoral Differences Dictate the Outcome of TGF-β Blockade on the Efficacy of Viro-Immunotherapy
Source: Cancer Res Commun. 2023 Feb 23;3(2):325–37. doi: 10.1158/2767-9764.CRC-23-0019 (PMC9973387; doi:10.1158/2767-9764.CRC-23-0019)
Supplement: Figure S4 — TGF-β blockade does not impair Reo&CD3-bsAb efficacy by decreasing T-cell influx or activation. [file crc-23-0019-s07.pdf]

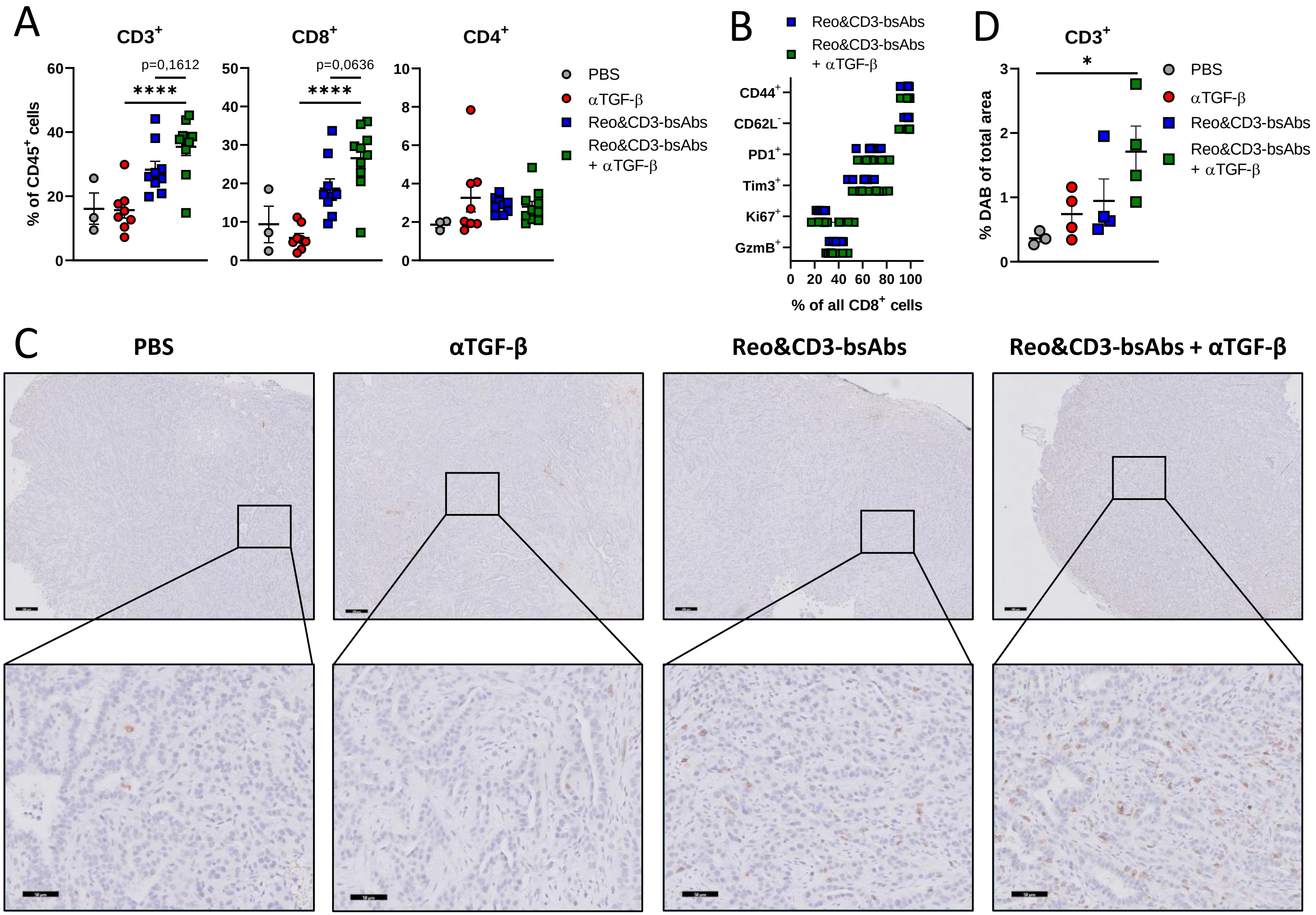

Figure S4. TGF-β blockade does not impair Reo&CD3-bsAb efficacy by decreasing T-cell influx or activation. (A) Frequency of $CD3^+$, $CD8^+$ and $CD4^+$ T cells within the total $CD45^+$ immune cell population in end-stage tumors after indicated treatments. **(B)** Expression of various markers on intratumoral CD8+ T cells after receiving Reo&CD3-bsAbs or Reo&CD3-bsAb + αTGF-β. **(C)** Immunohistochemistry staining for CD3 in representative tumors after indicated treatments. Scale bars represent 200 µm for overview and 50 µm for magnification, respectively. **(D)** Quantification of positive DAB signal in tumor coupes stained for CD3 after receiving indicated treatments. Data represent mean±SEM. Significance between groups in (A) and (D) was determined using an ordinary one-way ANOVA with Tukey's multiple comparisons test. Significance levels: ns=not significant, *p<0.05 and **p<0.01.
