# Supplementary material for: Intertumoral Differences Dictate the Outcome of TGF-β Blockade on the Efficacy of Viro-Immunotherapy
Source: Cancer Res Commun. 2023 Feb 23;3(2):325–37. doi: 10.1158/2767-9764.CRC-23-0019 (PMC9973387; doi:10.1158/2767-9764.CRC-23-0019)
Supplement: Figure S5 — CD8-specific TGF-β blockade does not impair the efficacy of Reo&CD3-bsAb therapy. [file crc-23-0019-s08.pdf]

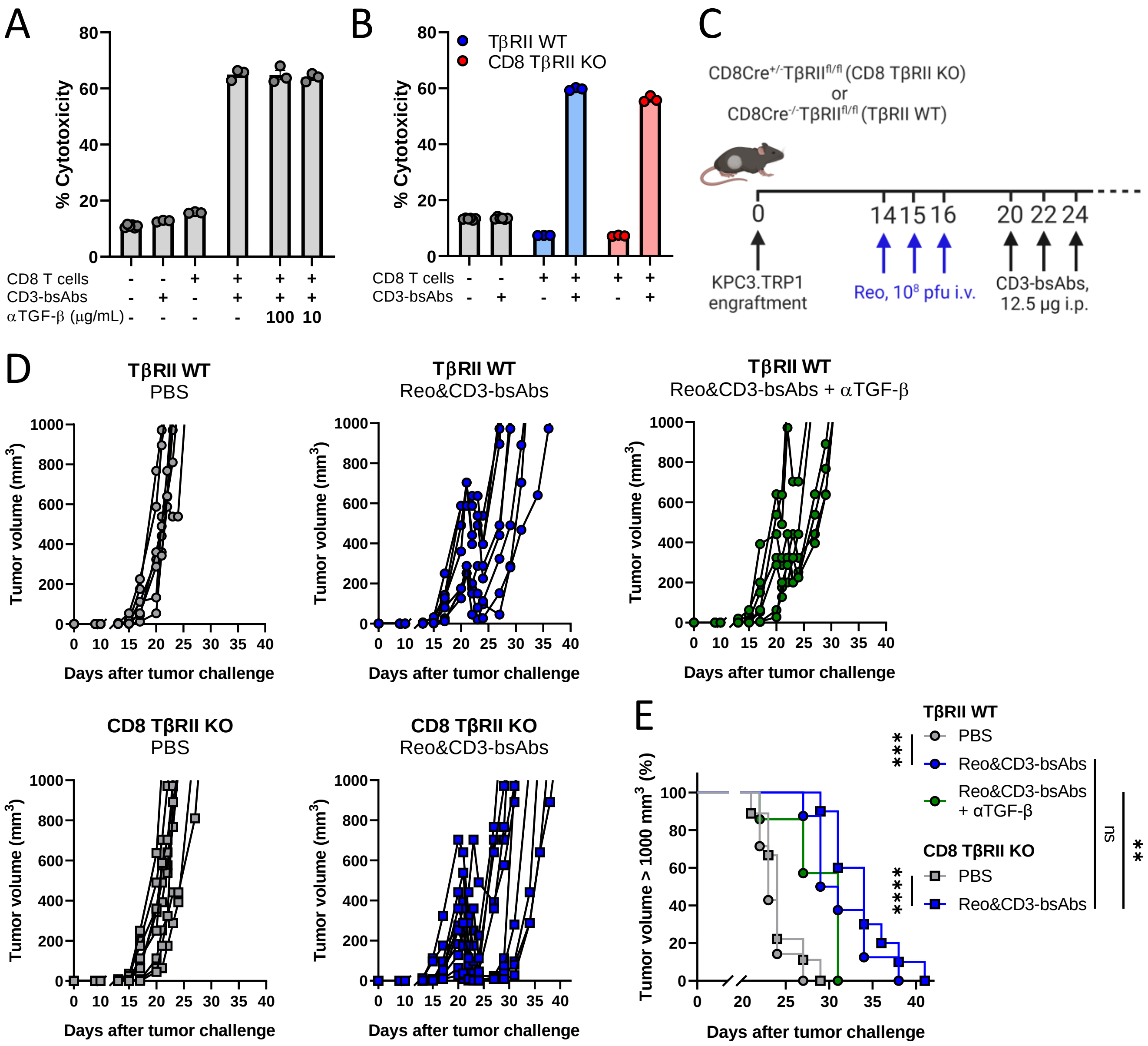

Figure S5. CD8-specific TGF-β blockade does not impair the efficacy of Reo&CD3-bsAb therapy. (A) Percentages of cytotoxicity of KPC3.TRP1 cells after in vitro co-culture with enriched CD8$^+$ T cells from naïve mice and CD3-bsAbs, in combination with TGF-β neutralizing antibodies. **(B)** Percentage of cytotoxicity of KPC3.TRP1 cells after in vitro co-culture with enriched CD8$^+$ T cells from TβRII WT or CD8 TβRII KO mice and CD3-bsAbs. Data represents mean±SEM of triplicates. **(C)** Overview of experiment described in (B-C). TβRII or CD8 TβRII KO mice (n=7-10/group) were subcutaneously engrafted with KPC3.TRP1 cells (1x10^5/mouse). Mice received Reo intravenously on day 14, 15 and 16 (10^8 plaque-forming units/injection) and received CD3-bsAbs intraperitoneally (12.5 μg/injection) on day 20, 22 and 24. Tumor growth was measured 3-5x/week. **(D)** Individual tumor growth curves of mice receiving indicated treatments. **(E)** Kaplan-Meier survival graphs of mice after indicated treatments. Log-rank tests were used to compare differences in survival in (E). Significance levels: ns= not significant, **p<0.01, ***p<0.001 and ****p<0.0001. Figure (C) was created with BioRender.com.
