# Supplementary material for: Intertumoral Differences Dictate the Outcome of TGF-β Blockade on the Efficacy of Viro-Immunotherapy
Source: Cancer Res Commun. 2023 Feb 23;3(2):325–37. doi: 10.1158/2767-9764.CRC-23-0019 (PMC9973387; doi:10.1158/2767-9764.CRC-23-0019)
Supplement: Figure S6 — Introduction of TRP1 expression on MC38.TRP1 cells allows killing via CD3-bsAbs. [file crc-23-0019-s09.pdf]

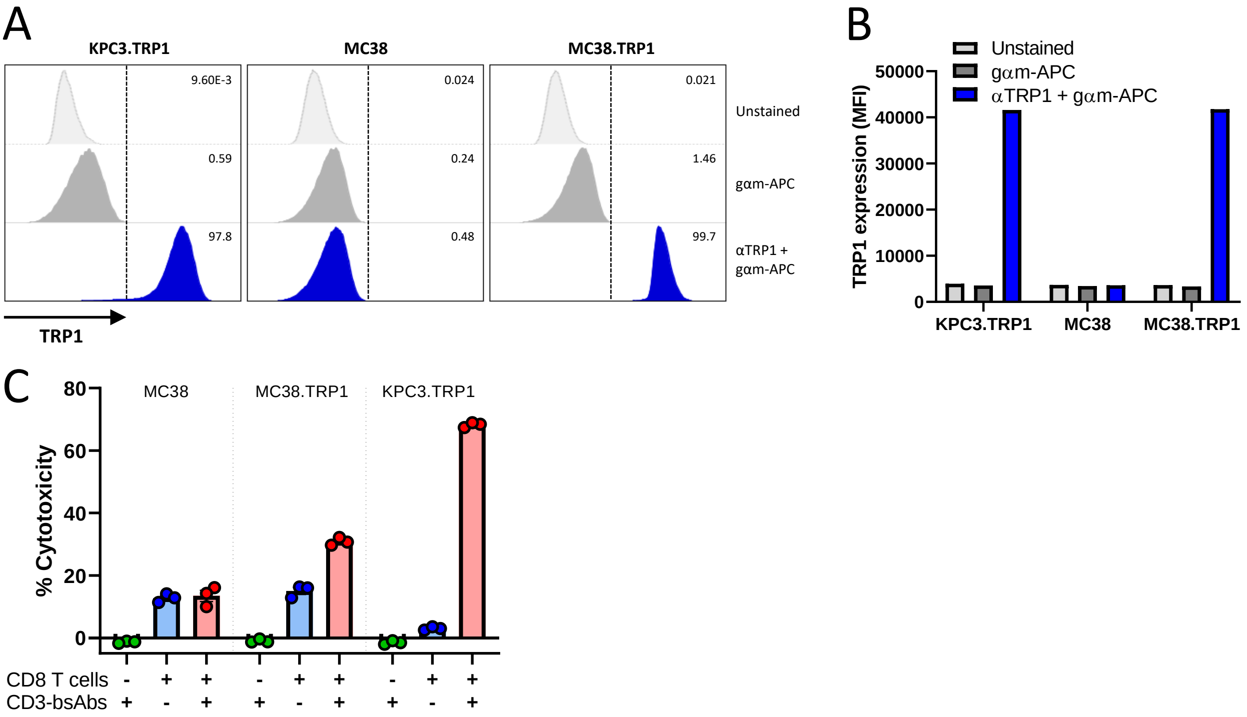

Figure S6. Introduction of TRP1 expression on MC38.TRP1 cells allows killing via CD3-bsAbs. (A) Percentage of TRP1 expression on MC38 cells after transfection and sorting, as measured by flow cytometry. Non-transfected MC38 cells are used as negative control and KPC3.TRP1 cells act as positive control. **(B)** Comparison of mean fluorescence intensity (MFI) of TRP1 signal between KPC3.TRP1 and MC38.TRP1. **(C)** Percentages of cytotoxicity of KPC3.TRP1, MC38 and MC38.TRP1 cells after in vitro co-culture with nylon-wool enriched T cells from naïve mice and CD3-bsAbs. Data represents mean±SEM of triplicates.
